# Identification of a coagulation-related classification and signature that predict disease heterogeneity for colorectal cancer and pan-cancer patients

**DOI:** 10.3389/fimmu.2025.1572701

**Published:** 2025-07-24

**Authors:** Junpeng Pei, Yuye Gao, Baojian Xing, Yongjiu Chen, Aiwen Wu

**Affiliations:** ^1^ State Key Laboratory of Holistic Integrative Management of Gastrointestinal Cancers, Unit III, Gastrointestinal Cancer Center, Peking University Cancer Hospital & Institute, Beijing, China; ^2^ Beijing Key Laboratory of Carcinogenesis and Translational Research, Unit III, Gastrointestinal Cancer Center, Peking University Cancer Hospital & Institute, Beijing, China; ^3^ Biliary Surgery (2nd General) Unit, Department of General Surgery, Shengjing Hospital of China Medical University, Shenyang, Liaoning, China

**Keywords:** coagulation, clustering, colorectal cancer, precision treatment, tumor microenvironment

## Abstract

**Background:**

While increased coagulation is linked to cancer progression, the specific roles of coagulation-related genes in colorectal cancer (CRC) have not been extensively studied. This research identified coagulation-related subtypes (CRSs) and evaluated a coagulation-related risk score for its prognostic value in CRC.

**Methods:**

CRC dataset from The Cancer Genome Atlas was analyzed to identify CRSs using nonnegative matrix factorization, which was validated across GSE39582 and pan-cancer datasets. A list of 285 coagulation-related genes was used to develop a risk signature via least absolute shrinkage and selection operator and multivariate Cox regression. We also assessed immune characteristics and treatment responses using single-sample gene set enrichment analysis, Tumor Immune Dysfunction and Exclusion, and immunophenoscore, and constructed an overall survival-related nomogram.

**Results:**

CRS analysis categorized pan-cancers, including CRC, into three clusters: C1 with poor immune infiltration but better prognosis, C2 with high immune activity and prolonged survival, and C3 marked by dense immunosuppressive cells correlating with poor outcomes. Drug sensitivity analysis showed distinct responses across CRSs, influencing treatment choices. We developed a coagulation-related risk score based on F2RL2, GP1BA, MMP10, and TIMP1, which stratified CRC patients by outcome and correlated with distinct patterns of immune infiltration and therapeutic response. A validated nomogram incorporating age, TNM stage, and risk score accurately predicted overall survival, while experimental validations confirmed the bioinformatics predictions regarding TIMP1’s role in CRC progression.

**Conclusions:**

A coagulation-based classifier effectively categorizes CRC and potentially other cancers, interacting significantly with the immune microenvironment to influence disease progression and treatment responsiveness. This approach offers valuable insights for personalized cancer therapy.

## Introduction

Coagulation is a dynamic and complex biological process that regulates the balance between clot formation and bleeding by triggering a cascade of reactions including the activation of coagulation factors and platelets (1, 2). According to theory, there is a close relationship between the coagulation system and malignancy. A number of cancer patients have coagulation abnormalities such as thrombosis and hemorrhage. The upregulation of procoagulant molecules is found in various cancers, especially tissue factor, which is associated with survival probability (3–6). Exposure of cellular procoagulant features during hemostatic activation can result in carcinogenesis at all cancer stages. Thereby, coagulation is associated with the development of cancer and the clinical prognosis for cancer patients. A deeper understanding of coagulation may help to identify potential biomarkers of cancer development and provide insight into new therapeutic options for cancer sufferers.

Colorectal cancer (CRC) ranks as the second leading cause of cancer-related deaths worldwide and stands as the third most prevalent form of cancer. Recurrence and metastasis of cancer still trap almost half of CRC patients, making patient survival extremely low (7, 8). Genetic heterogeneities within and between CRC tumours contribute to the tumour microenvironment (TME) and resistance to therapeutic intervention, which ultimately leads to tumour metastasis. Of note, hemostatic components such as D­dimer and fibrinogen are associated with clinical stage and can be potential prognostic markers for CRC mortality prediction, tumor progression, and therapeutic response (9–14). In CRC patients, activation of the coagulation system is often associated with an advanced stage of the tumour and a bad prognosis (10, 12, 15–18). Moreover, thrombotic events are common complications for CRC patients, indicating a hypercoagulable state (19). Therefore, gaining a deeper insight into the connection between the coagulation system and the CRC microenvironment is crucial.

**Figure 1:**
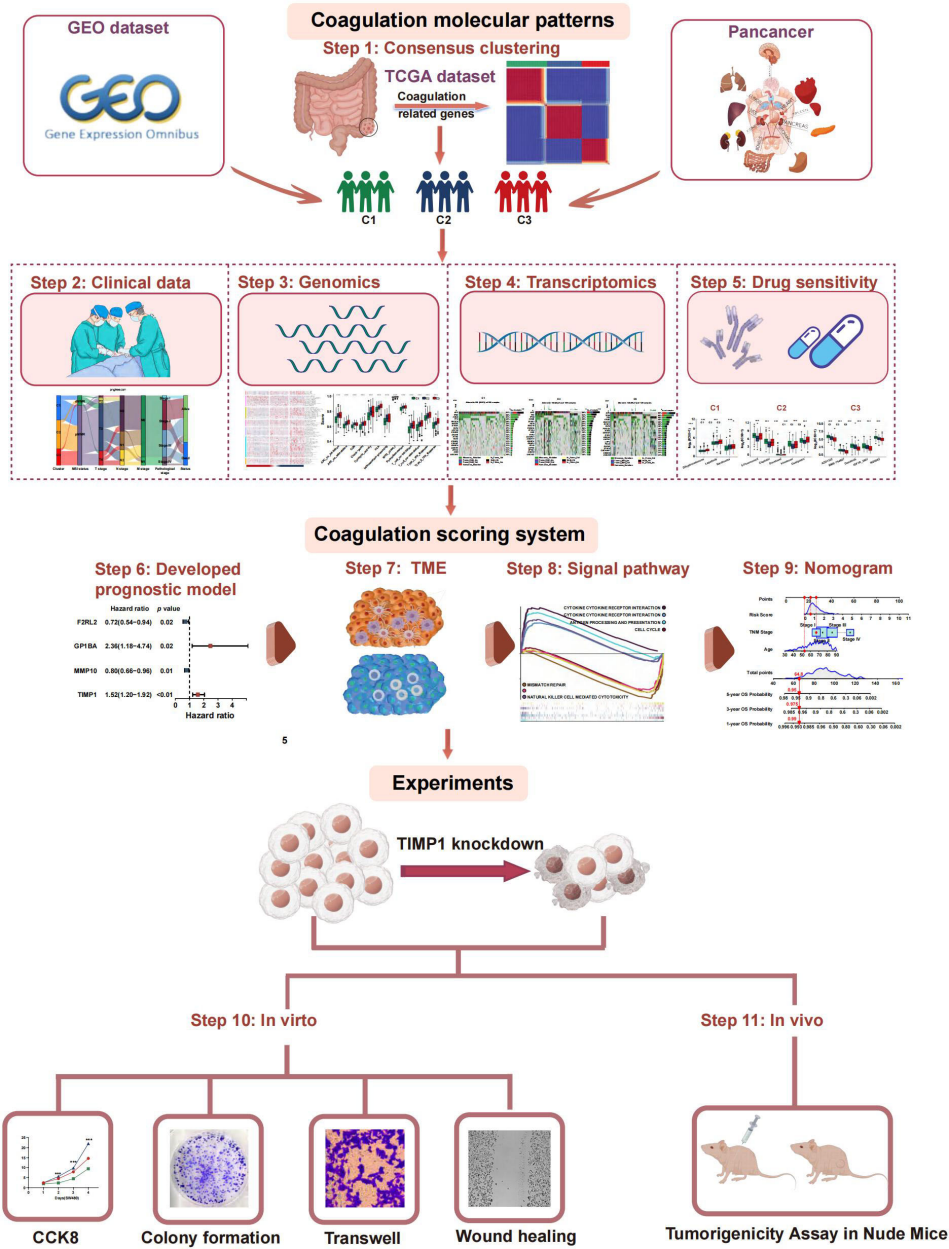
Workflow of the study.

To date, the Consensus Molecular Subtypes (CMS) framework classifies CRC into four groups—CMS1 (immune), CMS2 (canonical/WNT-MYC), CMS3 (metabolic), and CMS4 (mesenchymal)—and has been widely adopted for its biological interpretability (20). However, intra-subtype heterogeneity in patient prognosis, immune infiltration, and therapy response remains substantial, and CMS stability across pan-cancer cohorts is limited. Here, we introduce a novel coagulation-related subtype (CRS), derived from 285 coagulation-related genes (CRGs), and perform a head-to-head comparison with CMS. The prognostic value, TME, and treatment responses associated with these subtypes were evaluated. We validated subtype classification across an independent CRC cohort and extended the analysis to pan-cancer. Subsequently, we designed a coagulation-related risk score (CRRS) to predict the prognosis of CRC patients and assess the potential effectiveness of immunotherapy. Finally, *in vivo* and *in vitro* experiments evaluated coagulation-related hub genes by exploring coagulation associated gene expression patterns and the interconnection between coagulation and CRC. This approach provides for the development of more personalized and precise therapeutic strategies for treatment of CRC patients. The flow chart of the investigation is presented in **Figure 1**.

## Materials and methods

### Gathering and analysing data

Gene Expression Omnibus (GEO) (https://cancergenome.nih.gov/) and Cancer Genome Altas (TCGA) (https://portal.gdc.cancer.gov/) provided the clinical and transcripts per million (TPM) RNA-seq data for CRC. A pan-cancer study was conducted subsequently. Download GSE39582 (n = 585) from the GEO database to do an impartial queue verification. The pan-cancer subsets on XENA were the source of the pan-cancer transcriptome data, which were then normalised using log2 (X+1). 8473 samples were kept for further examination after non-tumor samples were eliminated. TCGA and GEO all included 285 CRGs, which were obtained via the gene set enrichment analysis (GSEA) (https://www.gsea-msigdb.org/gsea/index.jsp) and earlier research.

The gene list is displayed in the supplementary materials (**Additional File 10**, **Supplementary Table S1**). In addition, we downloaded alteration data including Copy number alteration (CNA) and Somatic mutation counts from the cbioportal online tool (https://www.cbioportal.org) (21). The R package “maftools” was used to analyze and visualize the somatic mutation data. It included many modules for analysis and visualization that are often employed in cancer genomic research (22). We also studied the relationships between clinical outcome and patients with or without DNA alteration. **Table 1** contains a list of the dataset profiles used in this investigation.

**Table 1:** The profile of datasets.

Set Type	Dataset	Sample Type	Number	Source
Training set	TCGA-COAD	tissue	480	TCGA
	TCGA-READ	tissue	167	TCGA
Validation set	GSE39582	tissue	585	GEO
	TCGA-PANCAN	tissue	8473	TCGA
Re-validation set	–	nude mouse	10	This study
	–	cell line	8	This study

### Non-negative matrix factorization clustering

First, using univariate COX regression to identify the genes related to prognosis for consensus clustering, we investigated the interaction connection between various CRGs. Utilizing the “NMF” package, we conducted a cluster analysis of all CRC samples based on CRGs, considering both survival status and duration. This approach effectively reduced dimensionality and facilitated the investigation of potential molecular subtypes (23). We employed the “brunet” method with 30 iterations for non-negative matrix factorization (NMF) clustering. To identify the optimal cluster count, we calculated four metrics—cophenetic, residuals, RSS, and silhouette—across a rank range of 2 to 10. Based on the cophenetic metric, we set the optimal cluster count to 3, forming three stable subtypes.

### Clinical outcome and TME landscape analysis

The “survminer” package was utilized to visualize survival differences across various groups. To illustrate the relationship between CRS and clinicopathological characteristics, a Sankey diagram was generated using the “ggalluvial” R package. Different coagulation patterns may indicate distinct tumor microenvironments. We assessed the immune score, stromal score, ESTIMATE score, and tumor purity for each sample using the ESTIMATE algorithm (24). The single-sample gene set enrichment analysis (ssGSEA) method was applied to quantify the enrichment levels of immune signatures based on the transcriptomic data (25). Previous study provided the marker genes, which include immune cell types, immune-related pathways, and activities (26). The “GSVA” R program was used to calculate the normalized enrichment score for each immunological sample in each gene set. We also used seven other algorithms including TIMER (27), CIBERSORT, CIBERSORT-ABS (28), QUANTISEQ (29), MCPCOUNTER (30), XCell (31), and EPIC (32) to ensure the robustness and the results of ssGSEA. Lastly, calculations were made to determine the variations in T-cell stimulators, immunological checkpoints, chemokines, and human leukocyte antigen (HLA). P-values less than 0.05 denoted statistical significance.

### Functional enrichment analysis

To identify the driving pathways of each coagulation-related subtype, we performed Gene Ontology (GO) and Kyoto Encyclopedia of Genes and Genomes (KEGG) enrichment analyses on the differentially expressed genes specific to each subtype. First, we converted gene symbols to Entrez IDs using the org.Hs.eg.db package in R. Subsequently, we conducted the analyses using the enrichGO() and enrichKEGG() functions from the clusterProfiler (v4.0) package, with the parameters set at pvalueCutoff = 1 and a significance threshold of p < 0.05.

### Prediction of immunotherapy and chemotherapy response

Using the “maftools” program, we were able to determine the tumor mutation burden (TMB) for each patient with colorectal cancer in the TCGA database. The tumor immune dysfunction and exclusion (TIDE) algorithm (http://tide.dfci.harvard.edu) was employed to assess three cell types that impede T-cell infiltration into tumors: interferon-gamma (IFNG), myeloid-derived suppressor cells (MDSC), and M2 subtypes of tumor-associated macrophages (TAM.M2). Additionally, it evaluated the exclusion of cytotoxic T lymphocytes (CTL) by immunosuppressive factors (Exclusion) and the dysfunction of CTL in tumor infiltration (Dysfunction) (33, 34). By comprehensively assessing these two immune evasion mechanisms, TIDE predicts the clinical response of patients to immunotherapy. Moreover, to evaluate the clinical response to programmed cell death protein 1 (PD-1) blockers and cytotoxic T-lymphocyte antigen 4 (CTLA-4) blockers and gauge the likelihood of tumor immune evasion, immunophenoscore (IPS) was utilized, which was downloaded from the Cancer Immunome Atlas (TCIA) database (https://tcia.at/home) (35). Tumor stemness was evaluated using RNAss, derived from mRNA expression, and DNAss, based on DNA methylation. The scores ranged from 0 to 1, with 1 indicating the highest level of undifferentiation. Drug sensitivity prediction was performed using the “pRRophetic” package (36), utilizing gene expression and drug sensitivity data from the Cancer Genome Project (CGP) as a training set (37). This approach employed ridge regression to forecast the sensitivity to various drugs.

### Development and validation of the coagulation-related risk score

The differential expressed CRGs among different clusters were screened using “limma” R package. Least Absolute Shrinkage and Selection Operator (LASSO) approach was used to focus the gene screening procedure (38). Subsequently, the Cox proportional hazards analysis identified genes significantly associated with coagulation. Subsequently, a CRRS was developed using the regression coefficients obtained from the multivariable Cox regression analysis along with the training dataset. The formula for the risk score was defined as follows:

Risk score = Σ Coefficient of (i)×Expression level of the gene (i), where “gene i” refers to the genes identified through the process.

The expression of gene (i) is the expression value of each candidate CRG (i) for each patient, and the coefficient of gene (i) is the regression coefficient of gene (i).

Harrell’s Concordance index (C-index) was a regularly used metric to verify the correctness of the regression model. Using the time-dependent receiver operating characteristic (ROC) curve, we also projected the survival rates of patients with colorectal cancer at 1, 2, 3, and 5 years. The ‘predict’ function in the survival R package was used to ascertain each patient’s risk score. Every patient was categorised into high- and low-risk categories based on the median value. Using analysis of risk score plots and survival curves within the training cohort, we evaluated the clinical value of the CRRS. Furthermore, the GEO cohort was used to confirm the prognostic accuracy of the CRRS.

### Nomogram development and assessment

The “rms” R package was employed to integrate independent prognostic factors—identified through both multivariate and univariate Cox analyses of the signature—and clinical variables to construct a nomogram. To validate the accuracy of the nomogram and the risk score, the C-index was calculated using the “pec” R package. Using the Hosmer-Lemeshow test, survival probability calibration curves for several years were produced. Additionally, we assessed the net benefit and clinical utility of the nomogram in comparison to a risk score using decision curve analysis (DCA).

### Analysis of single-cell RNA sequences

The Tumor Immune Single-Cell Hub (TISCH) analysis, which utilizes single-cell RNA sequencing data to provide detailed cell type annotations at the single-cell level, was used to evaluate data from the GSE146771 dataset to elucidate the elements of the tumor microenvironment (TME) (39, 40). We utilized the datasets derived from TISCH to calculate the expression of hub genes selected above in single cell populations.

### Quantitative real-time PCR and immunohistochemistry

To assess the protein expression levels of the signature gene in colorectal cancer and normal tissue samples, we analyzed IHC data and images obtained from the Human Protein Atlas (HPA, https://www.proteinatlas.org/). We cultured a normal human colon mucosal epithelial cell line (NCM460) along with seven colorectal cancer cell lines (SW480, HCT116, SW620, LS174T, HCT8, LoVo, and Caco2). Following RNA extraction from these cells, we performed quantitative real-time PCR (qPCR) to validate the expression of the signature gene. Total RNA was extracted using TRIzol reagent (#15596018, Invitrogen, Carlsbad, CA, USA) and subsequently reverse transcribed with a PrimeScript™ RT Reagent Kit (Cat#: E096-01A, Novoprotein, Shanghai, China). The qPCR was carried out following the TB Green Premix Ex Taq protocol (Novoprotein, Inc.), using specific primers. GAPDH was employed as the internal control, and the relative mRNA levels were determined by the 2-ΔΔCt method. Both technical and biological replicates were conducted in triplicate for each gene during RT-qPCR analysis. Additional details on the RNA molecules tested in the cell lines and the specific primers used can be found in **Additional File 10**, **Supplementary Table S2**.

### Cell culture and transfection

Human-derived CRC cell lines were obtained from the American Type Culture Collection (Manassas, VA, USA). These cells were maintained in either RPMI or DMEM media, both sourced from Gibco (Carlsbad, CA, USA), and supplemented with 10% fetal bovine serum (FBS). Regular passage of cells was carried out, and routine screenings for mycoplasma contamination were performed, ensuring cells were utilized only upon receiving negative test results.

The shRNA (Genepharma, China) targeting TIMP1 were recombined into lentiviral vectors to knockdown TIMP1, then CRC cells were selected by puromycin for 2 weeks after 72h infection. Stable knockdown cell lines were constructed and verified by q-PCR. The shRNA sequences used in this research are listed in **Additional File 10**; **Supplementary Table S3**.

### Cell proliferation and plate clone formation assays

The proliferation capacities of colorectal cancer cells were assessed using the cell counting kit-8 (CCK-8) assay (Biosharp, China). Cells were seeded into a 96-well plate at a density of 5 × 10^3^ cells per well, with 6 wells allocated for each group. Grouped as follows: HCT116 (NC, shTIMP1#1, shTIMP1#2), SW480 (NC, shTIMP1#1, shTIMP1#2). Following TIMP1 knockdown, the CCK-8 assay was conducted by adding 10μl of CCK-8 solution to each well, followed by a 1.5-hour incubation in a dark environment within an incubator. Subsequently, absorbance readings were obtained at a wavelength of 450 nm using a microplate reader (BD Biosciences, San Jose, CA, USA).

According to the same grouping method, we employed colony formation assays to assess cell proliferation capability. Cells were counted, diluted, and then seeded onto six-well plates, where they were cultured for 12 days. After the incubation phase, the colonies were treated with 4% paraformaldehyde for fixation and then stained using 0.5% crystal violet.

### Wound healing assay and transwell assay

Cells were plated into six-well dishes, and a wound line was created using a 200 μl disposable pipette tip. Once cell confluence reached around 80% to 90%, lines were etched across the cell layer. The plates were subsequently rinsed twice with PBS to eliminate any dislodged cells. Images of wound closure were taken at 0, 24, and 48 hours.

The cell migration capacity was evaluated using a transwell chamber (#3422, Corning, Cambridge, MA, USA). Cells were placed in the upper chamber containing 200 μl of serum-free medium at a concentration of 1.5 × 10^4^ cells per well. In the lower chamber, 700 μl of medium supplemented with 20% serum was added. Following a 36-hour incubation, the cells were fixed, stained with crystal violet, and the migrated cells were quantified using an inverted optical microscope.

### *In vivo* animal studies

All animal experiments were performed in strict accordance with the National Regulation of China for the Care and Use of Laboratory Animals. Male BALB/c nude mice, aged 6–8 weeks, were sourced from Beijing HFK Bio-technology Co. Ltd. (Beijing, China) and maintained in a specific pathogen-free (SPF) environment to develop the animal models. For each experimental group, 6 × 10^6^ treated CRC cells (sh-TIMP1 and sh-control) were collected and subcutaneously injected into the male BALB/c mice (n = 6). Tumor volumes were monitored every 72 hours using an electronic scale and vernier caliper, starting 6 days after tumor induction. After 27 days of treatment, all mice were humanely euthanized by cervical dislocation, and the tumors were harvested for further analysis.

### Statistical analysis

Bioinformatics analyses were performed using R software (version 4.2.1). A p-value of less than 0.05 was considered statistically significant. Differences between the two groups were assessed using either the paired two-tailed Student’s t-test or the Mann-Whitney-Wilcoxon test. For comparisons involving three groups, ANOVA or the Kruskal-Wallis rank-sum test was applied. The chi-square test was employed to analyze clinical characteristic differences.

## Results

### Landscape of genetic alteration profiles in CRC samples

**Figure 2:**
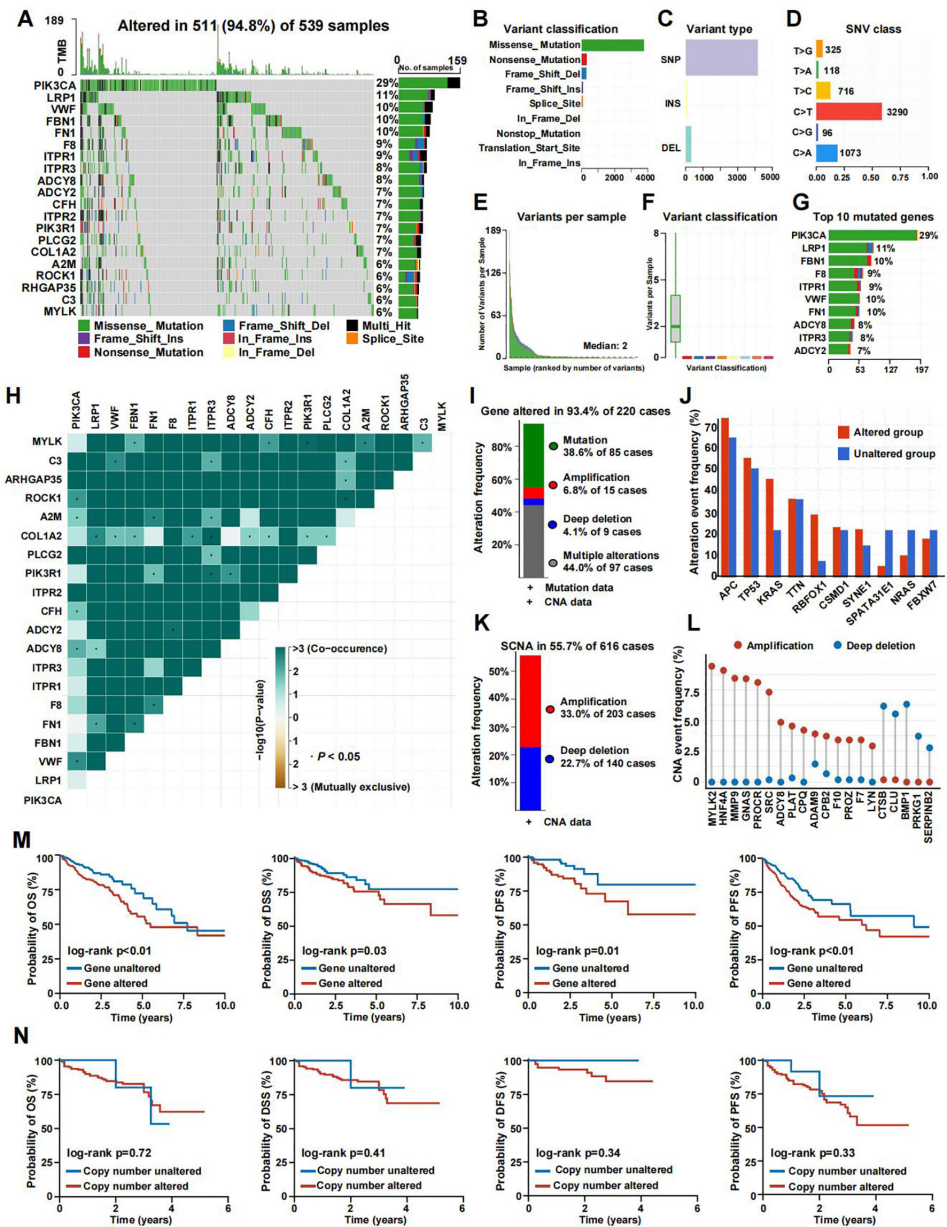
The genomic landscape of CRGs in CRC. **(A)** Mutation frequency of CRGs in TCGA-COADREAD. **(B–D)** According to different classification categories, missense mutation, SNP, and C>T mutation types accounted for a larger proportion. **(E)** Mutation burden in each sample. **(F)** The summary of the occurrence of each variant classification. **(G)** Top 10 mutated genes in CRC. **(H)** Mutual exclusion and synergistic heat maps of mutated genes in of CRGs in CRC. **(I)** Histogram of the proportion of gene alteration in CRC. **(J)** Gene alteration frequency of CRC patients in TCGA. **(K)** Histogram of the proportion of somatic copy number alteration in CRC. **(L)** The CNA frequency of CRGs. **(M)** Kaplan-Meier OS, PFS, DSS, and DFS curves between gene altered and gene unaltered group. **(N)** Kaplan-Meier OS, PFS, DSS, and DFS curves between copy number altered and copy number unaltered group.

We analyzed the mutation spectrum of 539 CRC patients using data from the TCGA database, focusing on 285 CRGs, including the 20 most frequently mutated ones. Mutations were present in 511 out of 539 samples (94.8%). The genes with the highest mutation frequencies in CRC were PIK3CA (29%), LRP1 (11%), VWF (10%), FBN1 (10%), and FN1 (10%) (**Figure 2A**). Upon categorizing the mutations, missense mutations emerged as the most prevalent type (**Figure 2B**). Single nucleotide polymorphisms (SNPs) were more frequently detected compared to insertions or deletions (**Figure 2C**), with C>T substitutions being the most prevalent type of single-nucleotide variant (**Figure 2D**). The median number of mutated bases per patient was two (**Figure 2E**). A box plot was used to show the frequency of different variant classifications (**Figure 2F**). By reanalyzing the data to account for the total number of mutations and the presence of multiple hits, we identified the top 10 most frequently mutated genes (**Figure 2G**). **Figure 2H** presents the mutual exclusivity and co-occurrence analysis for the top 20 mutated genes within the TCGA cohort. According to **Figure 2I**, CRG alterations were found in 93.4% of the 220 cases. **Figure 2J** displays the gene alteration frequencies among CRC patients, and **Figure 2K** reveals that 55.7% of CRC patients had at least one somatic CNA involving CRGs. Most of these genes exhibited high CNA frequency due to co-amplification rather than co-deletion (**Figure 2L**). Survival analysis indicated that patients with lower gene alteration frequencies had a better prognosis than those with higher frequencies (**Figure 2M**). However, no statistically significant difference was observed between the high and low CNA frequency groups (**Figure 2N**).

### Coagulation-related subtypes associated with prognosis and clinicopathologic features

**Figure 3:**
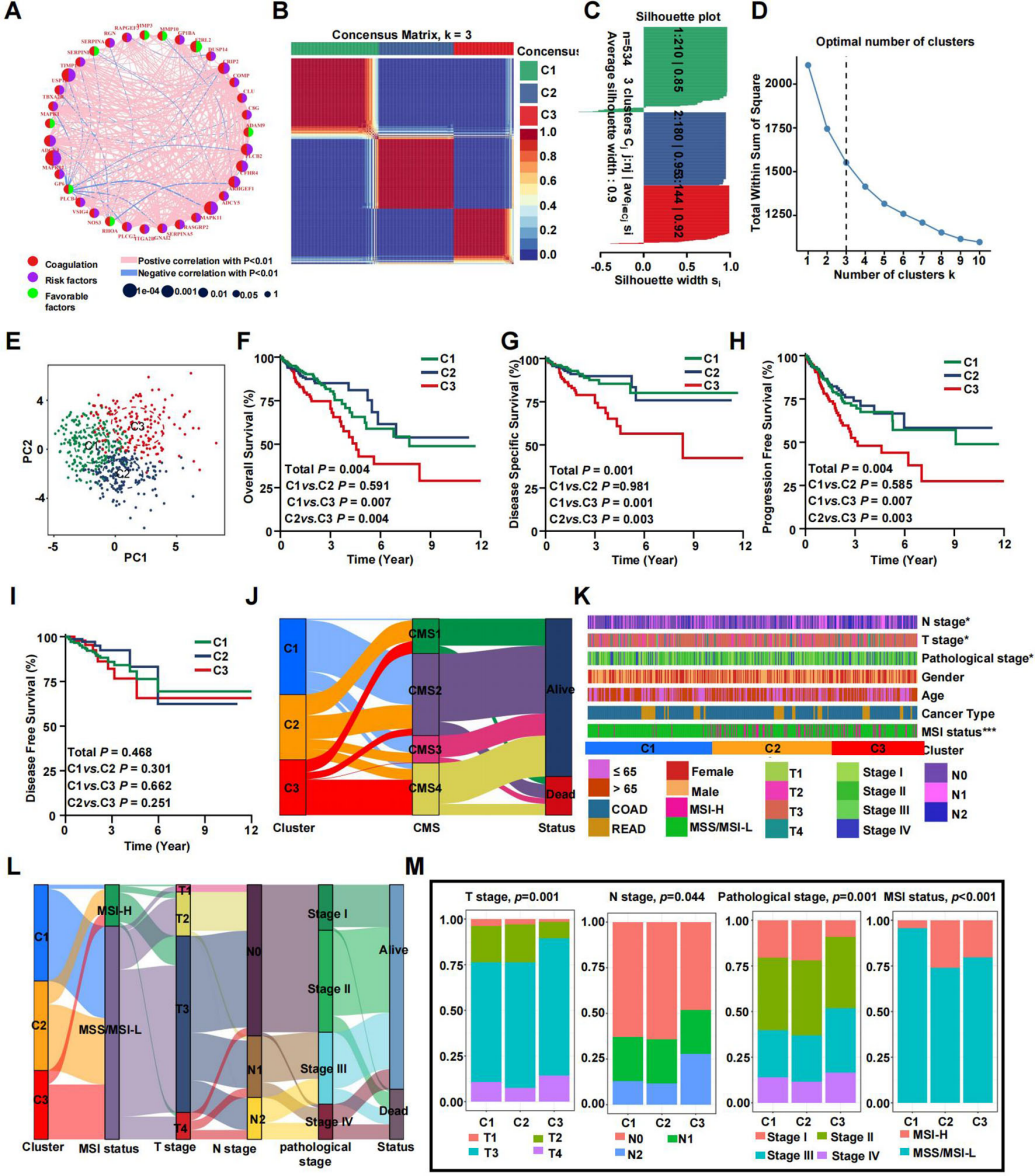
Prognostic association of CRGs classifications. **(A)** Network diagram showing the interaction of 35 CRGs in CRC. The size of the circles indicates the p-value of each gene on survival prognosis. purple represents risk factors, and green dots represent favorable factors. The thickness of the lines indicates the correlation values between genes. The red and blue lines represent positive and negative correlations of gene regulation, respectively. **(B)** The consensus clustering heat map visualizes the degree of segmentation for 35 genes in 534 samples. **(C)** The average silhouette width represents the coherence of clusters. **(D)** The optimal number of clusters. **(E)** Principal component analysis plots. **(F–I)** Kaplan-Meier overall survival **(F)**, disease-specific survival **(G)**, progress-free survival **(H)**, and disease-free survival curves **(I)**. **(J)** The correspondence between CRS, CMS and survival status. **(K)** Heatmap presenting the clinicopathologic features of these subtypes. **(L)** Sankey diagram showing the relationship between CRS, MSI status, T stage, N stage, TNM stage and status. **(M)** The distribution characteristics of different clinicopathological factors in three subtypes. (*p<0.05 and ***p<0.001).

A network was employed to visualize a comprehensive landscape of the intricate relationships among CRGs and the predictive accuracy for CRC patients (**Figure 3A**). Subsequently, we classified the CRC patients in the TCGA database into CRS utilizing the NMF algorithm (**Figure 3B**). According to the total within sum of squares (**Figures 3C, D**), the samples were divided into three subtypes. The contour widths of the three types were 0.85, 0.95, and 0.92 with an average silhouette width of 0.9. Principal component analysis (PCA) was conducted to compare the transcriptome expression of the various coagulation subtypes. In general, PCA results revealed that CRC samples were well-separated into three remarkably different subtypes, by CRGs (**Figure 3E**).

It is well-accepted that coagulation is firmly associated with the local growth as well as metastatic potential of CRC. Consistent with previous studies, survival analyses demonstrated a specific prognosis for various clusters. KM analyses revealed that patients in clusters C1 and C2 exhibited a prolonged overall survival (OS), while patients in cluster C3 had the worst outcomes (**Figures 3F–H**). However, there was no statistically significant difference in disease free survival (DFS) among patients in the three clusters (**Figure 3I**, P=0.468). **Figure 3J** demonstrates that C1 primarily corresponds to CMS2 and CMS3. In contrast, C2 showed no specific distribution pattern, and C3 was mainly related to CMS4. This alignment is in agreement with the characteristic features and prognostic indicators of CRS. Notable differences were found in clinicopathologic factors such as T stage, N stage, pathological stage, and microsatellite instability (MSI) status (**Figure 3K**). A Sankey plot illustrates the summarized distribution of various clinical features among the patients (**Figure 3L**). Furthermore, the proportion of higher T stage (T3/T4), higher N stage (N1/N2), and later pathologic stage (stage III/IV) CRCs was greatest in subtype C3, suggesting relationships among CRC subtypes, progression, and differentiation of CRC. In addition, patients in the C2 and C3 clusters were associated with more MSI status, which suggested a higher TMB (**Figure 3M**). By calculating the C-index and conducting DCA analysis, we found that our model’s predictive ability outperforms CMS molecular subtyping (**Supplementary Figures S1H–K**). The same trends were observed in the validation set (**Supplementary Figure S1**).

### Coagulation-based subtypes are associated with distinct TMEs

**Figure 4:**
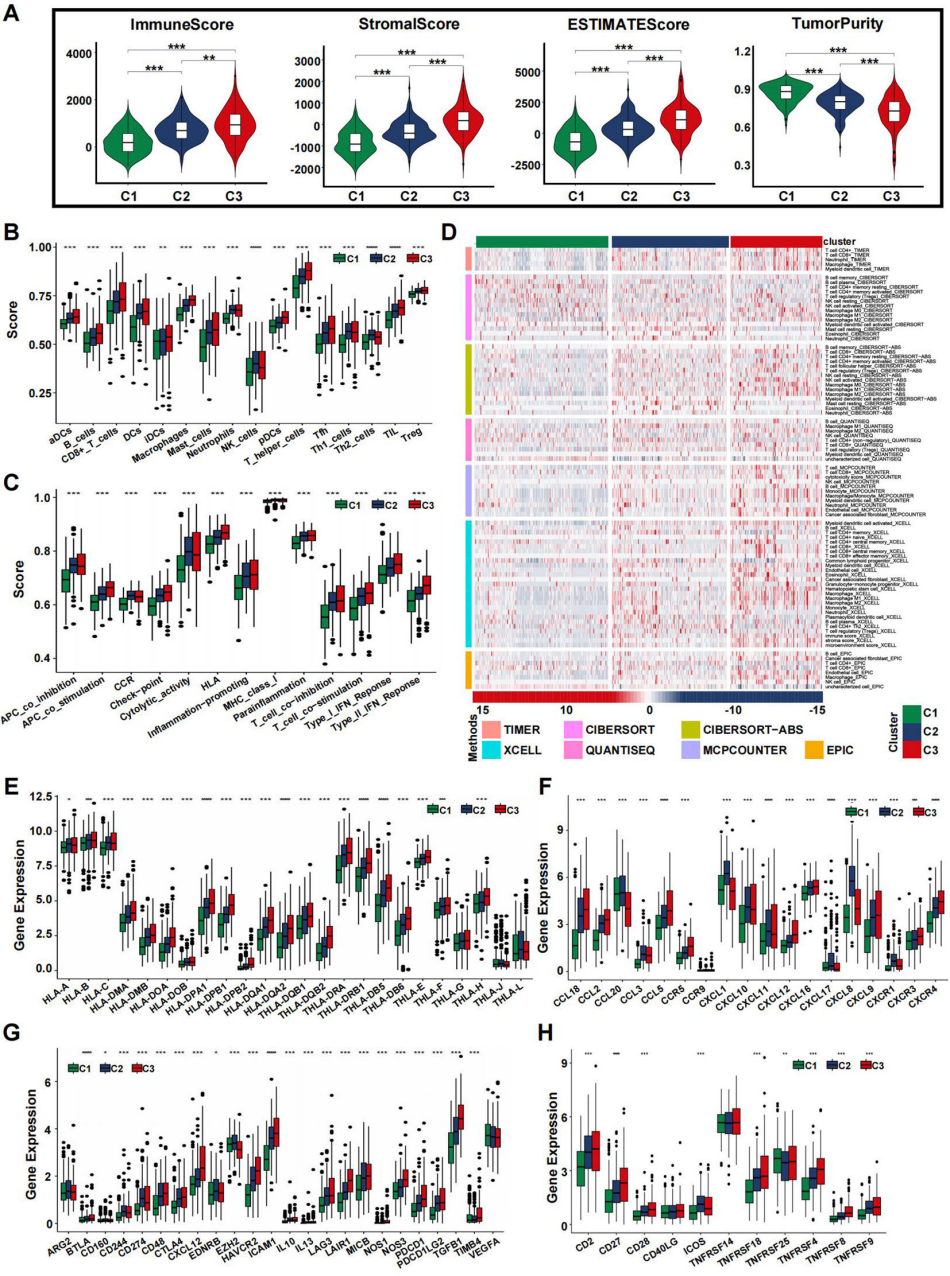
Immune landscape of CRS in the training set. **(A)** The violin plots display the immune score, stromal score, estimate score, and tumor purity score in the training cohort. **(B, C)** Immune cell infiltration **(B)** or functions **(C)** in the C1, C2 and C3 groups in the TCGA cohort. **(D)** Landscape of immune and stromal cell infiltration in the C1, C2 and C3 groups. Heatmap showing the normalized scores of immune and stromal infiltrations. **(E–H)** Boxplots representing the differential expression of HLA gene sets **(E)**, chemokines **(F)**, immune checkpoints **(G)**, and T-cell stimulators **(H)**. (*p<0.05, **p<0.01, ***p<0.001, and ****p<0.0001).

The coagulation system and CRGs are theoretically linked to the progression of malignant tumors and are also associated with the tumor microenvironment (TME). We investigated various cellular and acellular components within the TME. Overall, the immune score, stromal score, and estimate score gradually increased from cluster C1 to cluster C3, while tumor purity followed a similar upward trend (**Figure 4A**). These findings suggest that cluster C3 is characterized by a higher presence of immune cells, immune molecules, and stromal elements. Specifically, we assessed immune cell infiltration and immune activity across different subtypes using ssGSEA. The results indicated that cluster C3 was predominantly enriched with immunosuppressive cells and innate immune cells, including macrophages, immature dendritic cells, regulatory T cells, Th1 cells, and mast cells, in contrast to clusters C1 and C2. On the other hand, cluster C2 was rich in immunocompetent cells such as natural killer cells and Th2 cells (**Figure 4B**). Both clusters C2 and C3 showed significantly higher overall infiltration of immune-related pathways and increased immune activity compared to cluster C1, reflecting an environment of heightened immune activation (**Figure 4C**). An analysis of 29 immune signatures revealed that cluster C1 had the lowest abundance of immune-related cells and functions. Additionally, the distribution of infiltrating immune cells across the three clusters, as inferred by seven algorithms, demonstrated that most immune cells were more prevalent in clusters C2 and C3 (**Figure 4D**).

We further examined the expression of HLA family genes, chemokines, immune checkpoints, and T-cell stimulators across different CRS. Our findings revealed a significant upregulation of most HLA family genes in cluster C3 (**Figure 4E**). Chemokine expression analysis across the subtypes indicated a more intense inflammatory response in clusters C3 and C2 compared to C1 (**Figure 4F**). Additionally, immune checkpoint inhibitors displayed varying levels of expression, with uniform upregulation observed in the C3 cluster, suggesting that immune evasion might contribute to the poorer prognosis seen in patients with this subtype (**Figure 4G**). T-cell stimulators were also expressed at higher levels in clusters C2 and C3 (**Figure 4H**). In summary, our study identified distinct microenvironments associated with each subtype, which may underlie the observed differences in patient prognosis. The TME analysis results from the validation dataset were consistent with those from the training group (**Supplementary Figure S2**).

### Functional enrichment analysis of coagulation-related subtypes

Differentially expressed genes for the coagulation-related subtypes are listed in **Supplementary Table S2**. Overrepresentation analysis of GO and KEGG on the DEGs of C1, C2, and C3 subtypes revealed distinct pathway signatures (**Supplementary Tables S3**, **S4**).

C1 is characterized by adaptive immunity and antigen presentation. Key GO terms include antigen processing and presentation via MHC class II, humoral immune response, and chemotaxis. Enriched KEGG pathways include Antigen processing and presentation, PI3K–Akt signaling pathway and Complement and coagulation cascades. C2 exhibits an innate immune and inflammation-driven profile with matrix remodeling. Prominent GO terms are granulocyte migration, neutrophil chemotaxis, defense response to bacterium, and collagen catabolic process. Top KEGG pathways include Chemokine signaling pathway, TNF signaling pathway, and NF-κB signaling pathway. C3 is marked by complement activation and platelet–matrix interactions. Key GO terms include complement activation (classical pathway), collagen fibril organization, and extracellular structure organization. Enriched KEGG pathways include Complement and coagulation cascades and ECM–receptor interaction. These enriched GO terms and KEGG pathways highlight the unique immunological and coagulation profiles of each subtype.

### Somatic mutation landscape and prediction of clinical treatment efficacy

**Figure 5:**
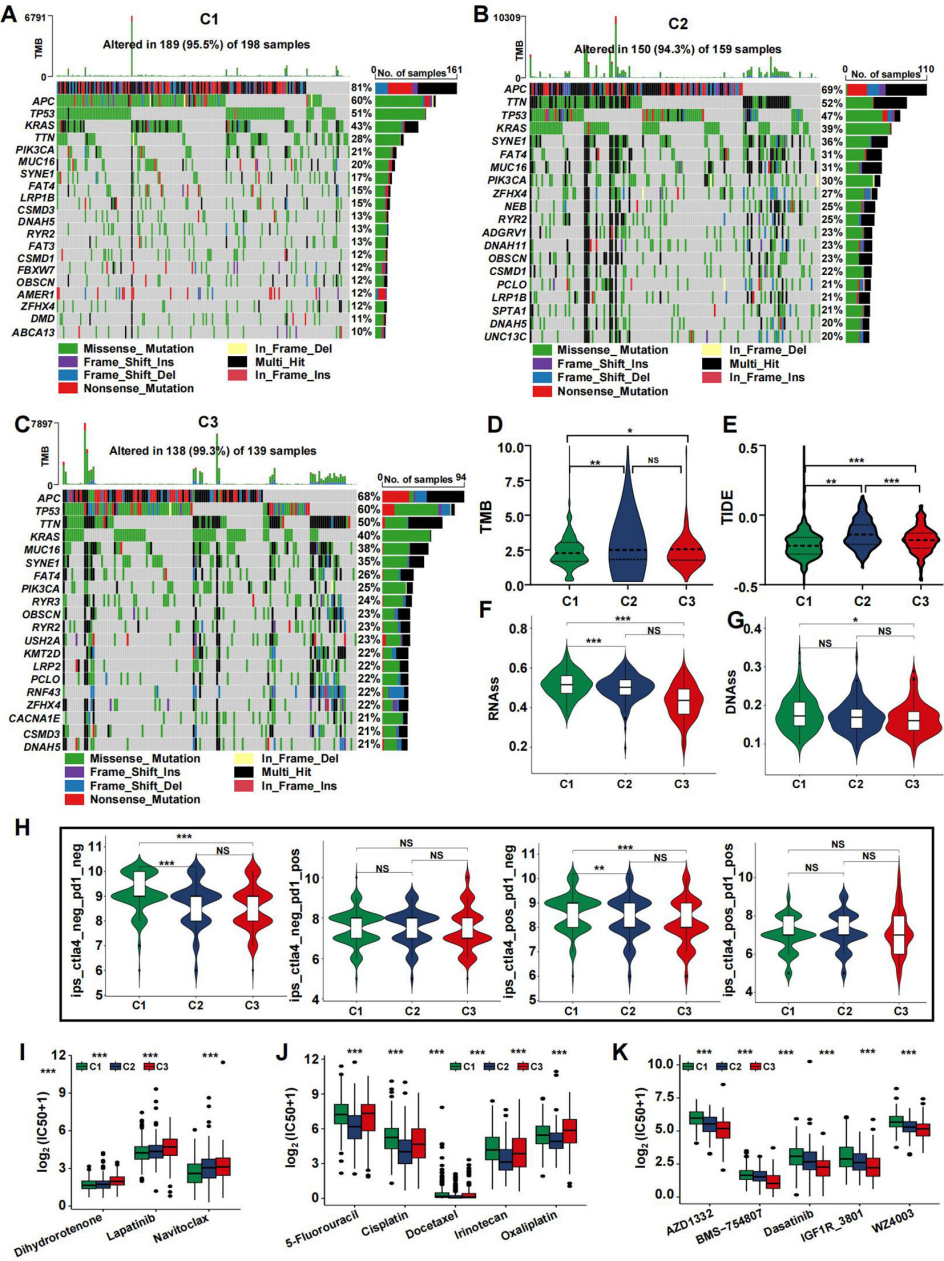
Mutation landscape of CRS and drug sensitivity. **(A–C)** Top 20 mutated genes in the C1 **(A)**, C2 **(B)**, and C3 **(C)** in the TCGA cohort. **(D-H)** Violin plots presenting the TMB score **(D)**, TIDE score **(E)**, RNAss **(F)**, DNAss **(G)**, and IPS scores **(H)** in CRSs. **(I–K)** drug sensitivity analysis for C1 **(I)**, C2 **(J)**, and C3 **(K)**. (*p<0.05, **p<0.01, and ***p<0.001).

The frequency of somatic mutations varied among the different subtypes. Although APC was the most frequently mutated gene, the rate of APC mutations differed across subtypes. Specifically, the C2 and C3 subtypes exhibited reduced APC mutation frequencies, with 68% and 69% of the total mutations, respectively, compared to 81% in the C1 subtype (**Figures 5A–C**). To determine the relationship between coagulation subtypes and immunotherapeutic efficacy, we assessed several indicators including TMB, TIDE, and immunophenoscore (IPS). Our analysis revealed that patients in clusters C3 and C2 had the highest TMB values, which corresponded to greater MSI status (**Figure 5D**). It is well established that a lower TIDE score is associated with a better clinical response to immunotherapy. As shown in **Figure 5E**, the TIDE score was highest in cluster C2, whereas cluster C1 had the lowest score. To further explore the relationship between coagulation subtypes and response to CTLA-4 and PD-1 blockers, we calculated the IPS for CRC patients. The results indicated that cluster C1 was significantly associated with a better response to these immunotherapies (**Figure 5H**), suggesting that patients in this group had the most favorable response to treatment. Additionally, tumor stemness was evaluated using RNAss and DNAss, revealing that tumors in the C1 subtype had the highest levels of stemness (**Figures 5F, G**).

Using the pRRophetic R package, we calculated the IC50 values for key chemotherapeutic agents and targeted therapies across CRC samples. The results indicated that most drugs exhibited a potent cell-killing effect on the C2 subtype, which may contribute to its favorable prognosis. Several widely used CRC treatments, including 5-Fluorouracil, Irinotecan, and Oxaliplatin, showed significantly different effects in the C2 group (**Figure 5J**, p<0.05). Conversely, the C1 subtype had lower IC50 values for Lapatinib, Dihydrorotenone, and Navitoclax (**Figure 5I**). The C3 subtype demonstrated reduced IC50 values for AZD1332, BMS−754807, Dasatinib, IGF1R_3801, and WZ4003 (**Figure 5K**).

### Pan-cancer analysis

**Figure 6:**
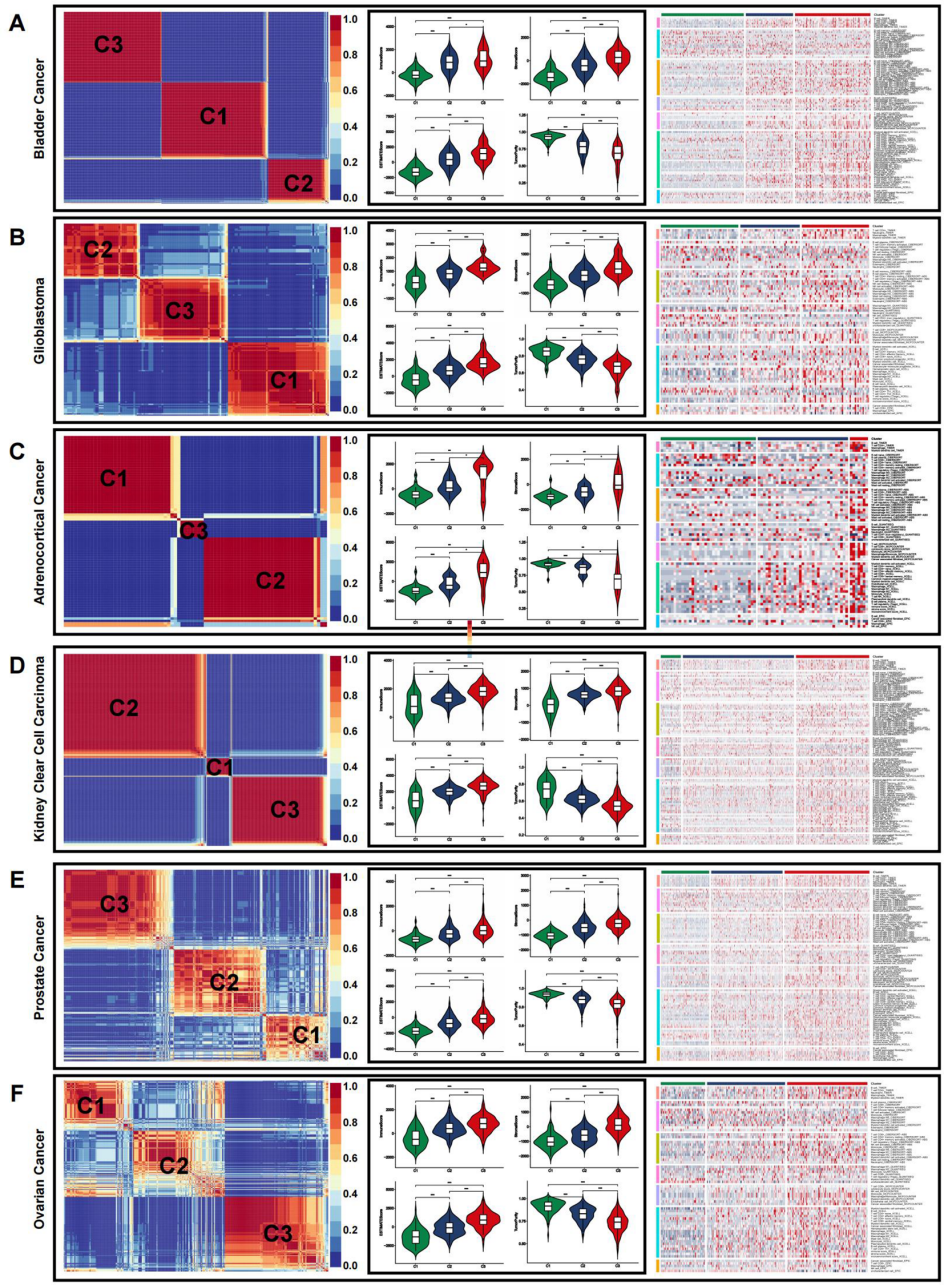
Immune microenvironment of CRSs in pan-cancer. **(A–F)** The immune microenvironment in representative cancer: bladder cancer **(A)**, glioblastoma **(B)**, adrenocortical cancer **(C)**, kidney clear cell carcinoma **(D)**, prostate cancer **(E)**, and ovarian cancer **(F)**. (*p<0.05, **p<0.01, and ***p<0.001).

Given the link between coagulation and various cancer types, we explored the applicability of the previously mentioned classification in other cancers. The algorithm was applied to categorize cancers, using TCGA-COADREAD as the training dataset. All solid tumors were successfully classified into three distinct subtypes with clear boundaries (**Figure 6**, **Supplementary Figures S3**–**S6**).

We then employed the ESTIMATE algorithm to evaluate the overall TME status across different cancer types. In most cases, the immune landscape mirrored that observed in CRC. The highest estimate, stromal, and immune scores were observed in the C3 subtype, indicating a higher abundance of immune cells and stromal components. Conversely, C3 exhibited the lowest tumor purity, which was inversely correlated with the estimate score. Immune infiltration analysis using multiple algorithms revealed significant differences in the levels of various immune cells among the pan-cancer CRS (**Figure 6**, **Supplementary Figures S3**–**S6**).

### Development and validation of a coagulation-related prognostic signature

**Figure 7:**
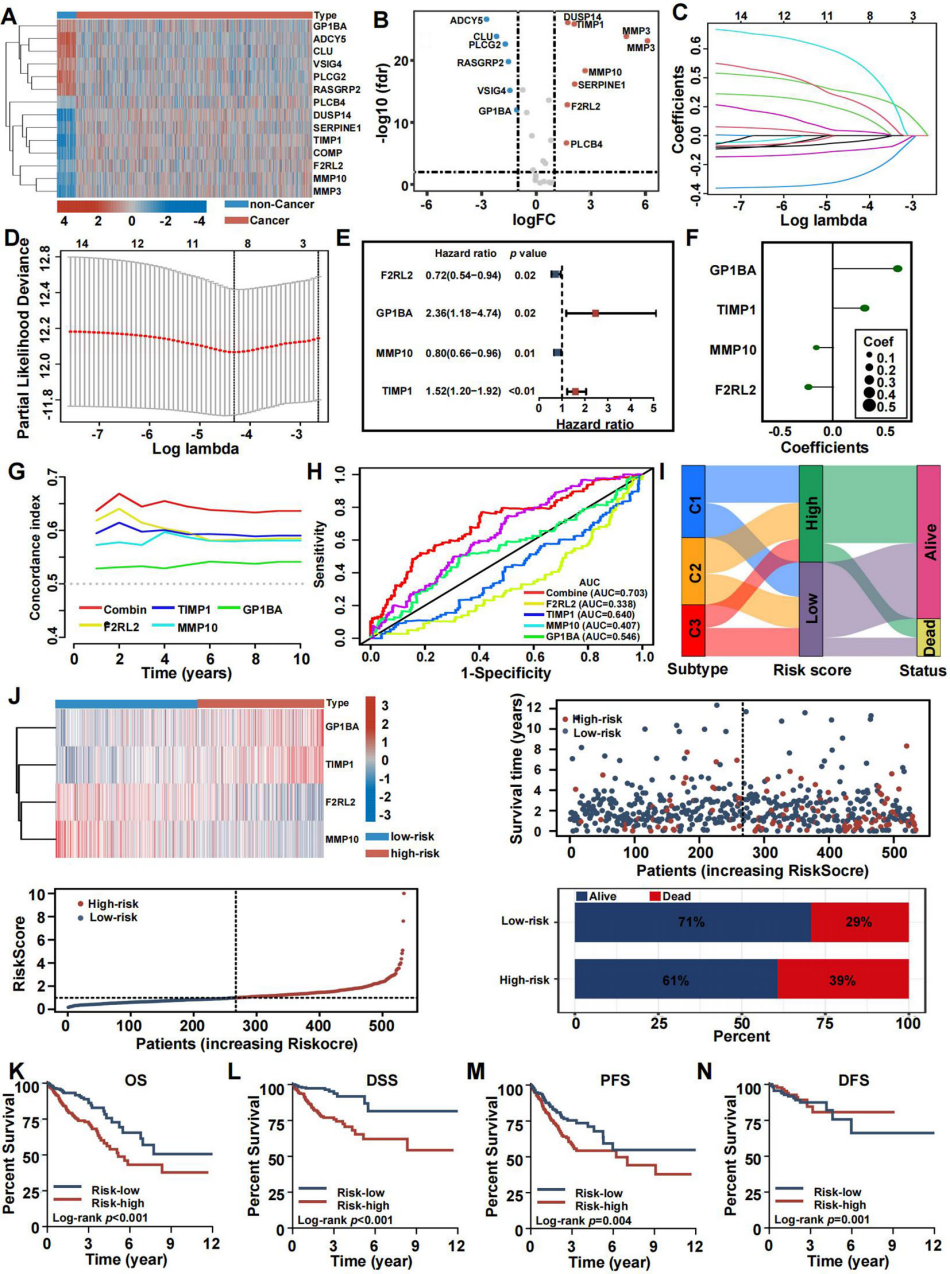
Construction and validation of the coagulation-related prognostic signature in training set. **(A)** Heatmaps of the signatures in the screening set after differential analysis. **(B)** Volcano plot of signatures after differential analysis. **(C, D)** Lasso Cox analysis of 14 differential expressed CRGs. **(E)** Multivariate Cox analysis uncovered 4 CRGs associated most with overall survival. **(F)** The coefficient of the 4 genes identified by Cox analysis. **(G)** Time-dependent C-index plot for the risk score and individual genes. **(H)** The AUC assess the accuracy of the risk score. **(I)** Sankey plot summarized the relationships among the clusters, risk score and survival status. **(J)** Survival status and risk score of the two risk groups. **(K–N)** Kaplan-Meier OS **(K)**, DSS **(L)**, PFS **(M)**, and DFS **(N)** curves for patients with high- or low-risk scores in TCGA cohort.

To investigate the connection between coagulation and colorectal cancer (CRC), we analyzed the expression levels of CRGs in both cancerous and non-cancerous groups (**Figure 7A**). Out of 285 CRGs, 14 differentially expressed genes (DEGs) were identified, as visualized in a volcano plot (**Figure 7B**). We analyzed the differentially expressed CRGs by applying LASSO regression in order to minimize the potential over-fitting problem and as well to narrow optimal prognostic signatures for the 285 CRGs. From the analyses, eight genes were selected with the optimal adjustment parameter (λ) (**Figures 7C, D**). In the final analysis, multivariate Cox regression identified four prognosis-related genes among the CRGs: F2RL2 (P=0.02), GP1BA (P=0.02), MMP10 (P=0.01), and TIMP1 (P<0.01). A risk-score model was constructed using the following equation: risk score = (0.9015) × GP1BA + (0.4606) × TIMP1 + (-0.2331) × MMP10 + (-0.3357) × F2RL2 (**Figures 7E, F**). Time-dependent C-index curves were generated for various factors, revealing that the combined model had the highest C-index compared to individual variables (**Figure 7G**). Additionally, the combined model displayed the largest area under the ROC curve (AUC) relative to other single markers, highlighting the risk-score model’s strong predictive and prognostic capabilities (**Figure 7H**). Patients were stratified into high-risk and low-risk groups based on the median risk score. A Sankey diagram visually depicted the relationships between CRS, risk scores, and patient outcomes (**Figure 7I**). Risk heat maps from the training cohort demonstrated that as risk increased, GP1BA and TIMP1 expression levels rose, indicating high-risk genes, while F2RL2 and MMP10 expression levels decreased, indicating low-risk genes. The high-risk group demonstrated a significantly higher mortality rate compared to the low-risk group (**Figure 7J**). Kaplan-Meier analysis further validated the prognostic accuracy of the risk model, revealing that higher risk scores were linked to poorer OS, progression-free survival (PFS), DFS, and disease-specific survival (DSS) in the TCGA training cohort (**Figures 7K–N**). These results were also confirmed in the GEO cohort (**Supplementary Figure S7**).

### Risk-score model somatic mutation and TME landscape

**Figure 8:**
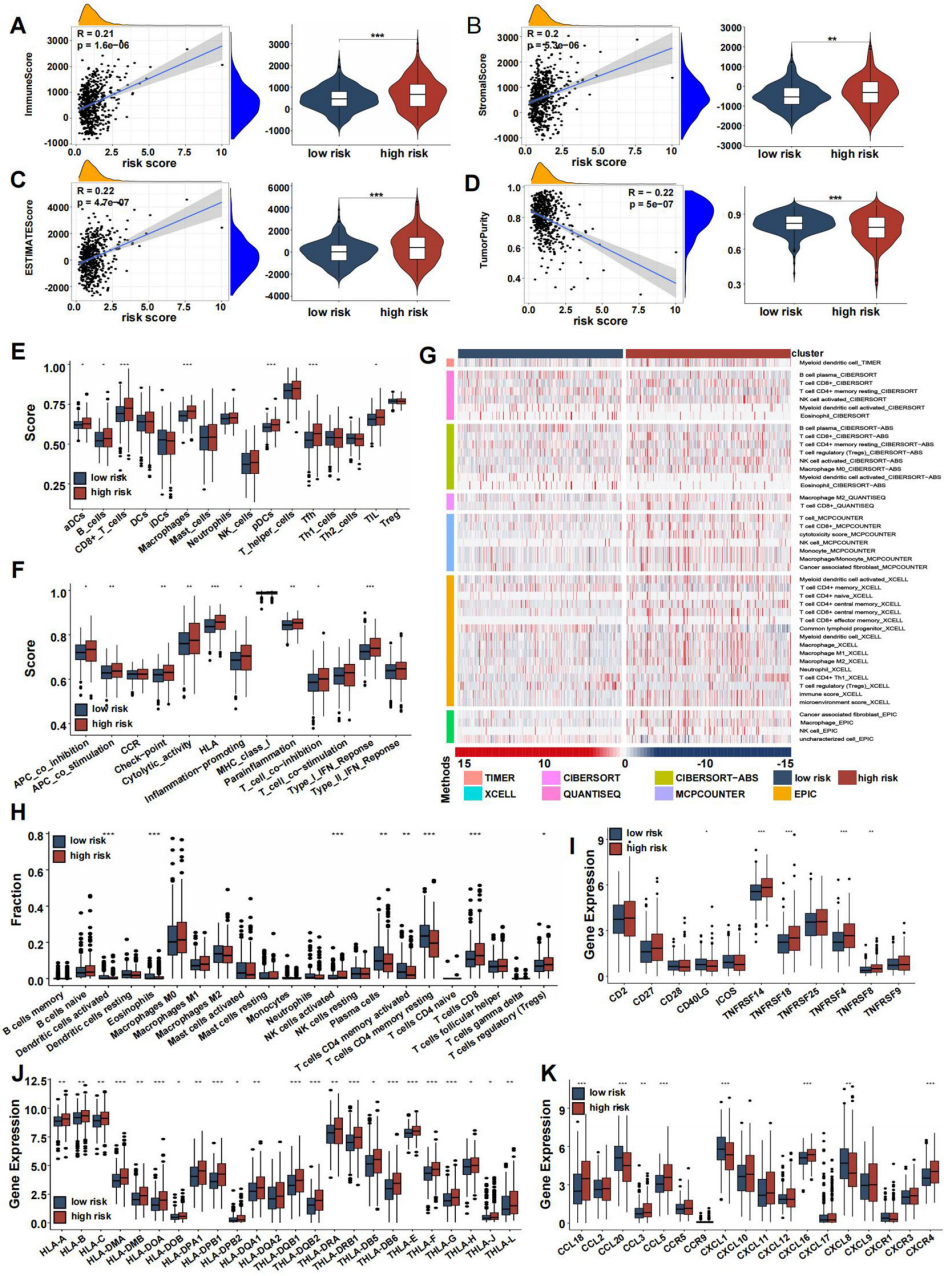
Immune association of coagulation-related risk score. **(A–D)** Violin plots comparing the immune score **(A)**, stromal score **(B)**, ESTIMATE score **(C)**, and tumor purity **(D)** between high- and low-risk groups. **(E)** Box plot comparing scores for 16 immune cell types between high- and low-risk groups. **(F)** Box plot comparing scores for 13 immune-related functions high- and low-risk groups. **(G)** Verification of ssGSEA results by seven other algorithms, namely TIMER, CIBERSORT, CIBERSORT-ABS, QUANTISEQ, MCPCOUNTER, XCell, and EPIC. **(H–K)** Boxplots representing the fraction of immune cell types **(H)**, differential expression of T-cell stimulators **(I)**, HLA gene sets **(J)**, chemokines **(K)**. (*p<0.05, **p<0.01, and ***p<0.001).

After dividing TCGA CRC patients into high-risk and low-risk groups based on CRRS, we analyzed the tumor microenvironment (TME) in both categories. Risk scores showed a positive correlation with the immune score (**Figure 8A**), stromal score (**Figure 8B**), and estimate score (**Figure 8C**), while being negatively correlated with tumor purity (**Figure 8D**). Immune infiltration analysis further revealed a higher abundance of immune-related cells, including activated dendritic cells, B cells, CD8+ T cells, macrophages, plasmacytoid dendritic cells, follicular helper T cells, and tumor-infiltrating lymphocytes in the high-risk group compared to the low-risk group (**Figure 8E**), with an enhanced immune response also observed in the high-risk group (**Figure 8F**).

The distribution of infiltrating immune cells between the two risk groups, evaluated using seven different databases, showed an increase in immunocytes such as macrophages, NK cells, CD4+ T cells, and CD8+ T cells in the high-risk group, while CD4+ memory T cells had reduced infiltration (**Figure 8G**). Box plots highlighted differences in immune cell infiltration, indicating that activated dendritic cells, eosinophils, plasma cells, and activated/resting CD4+ memory T cells were more prevalent in the low-risk group, whereas NK cells, activated T cells, CD8+ cells, and regulatory T cells (Tregs) were more abundant in the high-risk group (**Figure 8H**). T-cell stimulator expression was generally higher in the high-risk group, except for CD40LG (**Figure 8I**).

One critical step in the adaptive antitumor response is the presentation of tumor antigens by HLA molecules to activate CD8+ T cells. Due to the importance of HLA, we compared its expression between the two groups, finding significantly higher levels in the high-risk group (**Figure 8J**). Chemokine expression levels varied between the groups, as shown in **Figure 8K**. In both the screening and validation cohorts, heatmaps highlighted 29 immune-related gene sets, immune scores, stromal scores, estimate scores, and tumor purity (**Supplementary Figures S8A, B**), with validation dataset results consistent with the training set (**Supplementary Figures S8C–I**).

**Figure 9:**
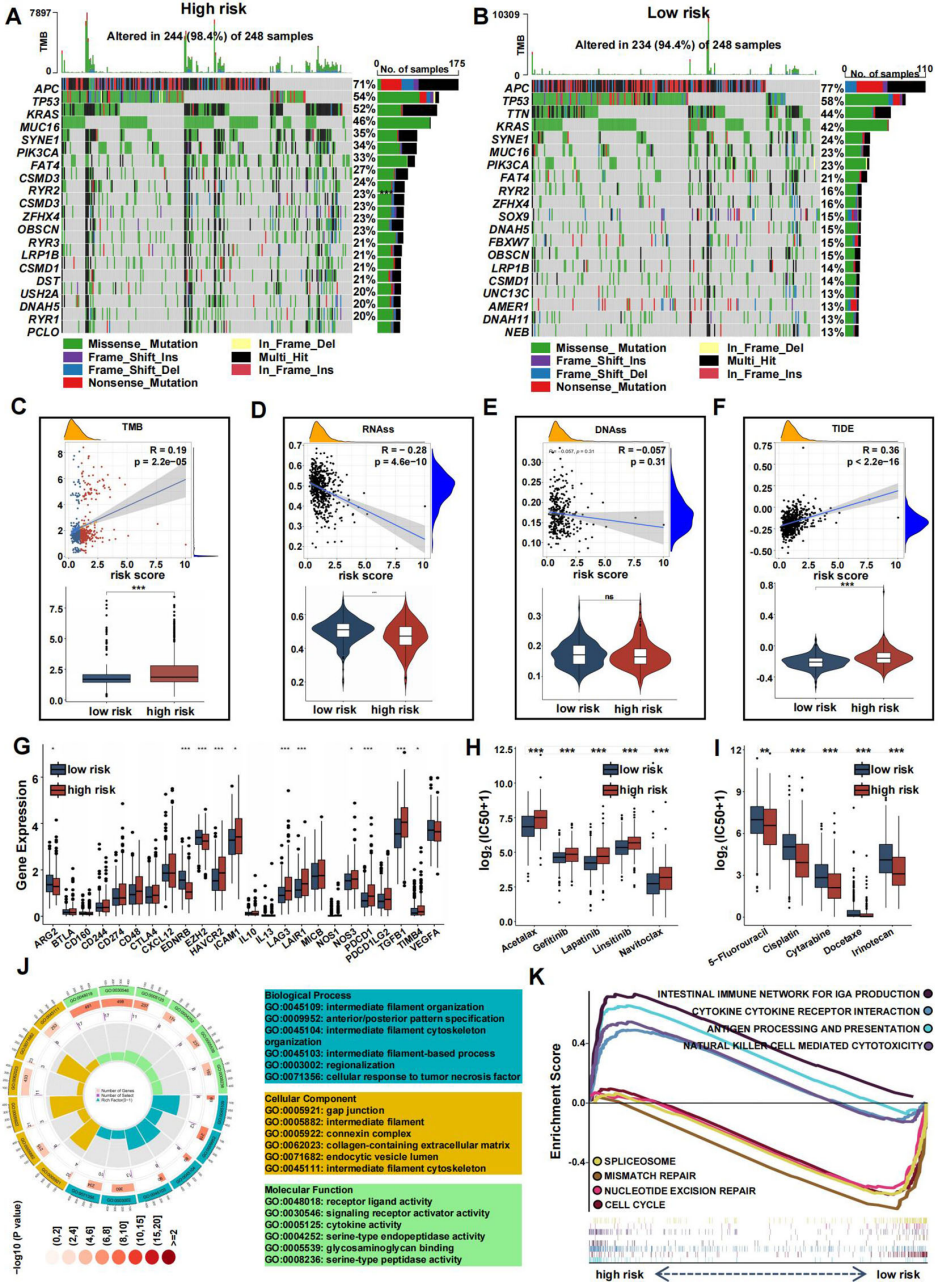
Mutation landscape and drug sensitivity of risk score. **(A, B)** Top 20 mutated genes in the high- **(A)** and low-risk group **(B)**. **(C–F)** Violin plots presenting the TMB score **(C)**, RNAss **(D)**, DNAss **(E)**, and TIDE score **(F)**. **(G)** The differential expression of checkpoint genes in the two risk groups. **(H-I)** Drug sensitivity between the low- **(H)** and high-risk **(I)** group. **(J)** GO analysis of DE-CRGs in terms of biological process, cellular component and molecular function. **(K)** GSEA of the DE-CRGs showing the different pathways in the low-risk group and in the high-risk group. (*p<0.05, **p<0.01, and ***p<0.001).

To clarify distinctions between high- and low-risk groups, we analyzed gene mutations in each group. The high-risk group showed a higher TMB compared to the low-risk group. We analyzed the top 20 genes with the highest mutation frequencies in each group. APC (77% vs. 71%) and TP53 (58% vs. 54%) had higher mutation frequencies in the high-risk group, whereas KRAS (46% vs. 42%) had a lower mutation frequency. Furthermore, TTN displayed a significantly higher mutation rate in the high-risk group compared to the low-risk group (**Figures 9A, B**). A significant correlation between risk score and TMB was observed, with TMB showing a positive correlation and RNAss a negative one (**Figures 9C, D**). No significant correlation was found between DNAss and risk score (**Figure 9E**). Additionally, the TIDE score was higher in the high-risk group, suggesting a poorer response to immune checkpoint inhibitors (**Figure 9F**). Several commonly mutated checkpoint genes, including PDCD1, were notably elevated in the high-risk group (**Figure 9G**).

The high-risk group also showed greater sensitivity to chemotherapeutics such as 5-Fluorouracil, Cisplatin, Cytarabine, Docetaxel, and Irinotecan, while the low-risk group was more responsive to drugs like Acetalax, Gefitinib, Lapatinib, Linsitinib, and Navitoclax (**Figures 9H, I**).

To explore the differences between the high-risk and low-risk groups, we conducted a differential analysis to identify DEGs. These DEGs were then subjected to GO analysis, which revealed significant enrichment in biological processes related to regionalization, collagen-containing extracellular matrix within cellular components, and activities such as signaling receptor activation and receptor-ligand interactions in molecular functions (**Figure 9J**). Additionally, gene set enrichment analysis (GSEA) showed that pathways associated with cancer progression, including mismatch repair, nucleotide excision repair, spliceosome, and cell cycle, were more active in the low-risk group (**Figure 9K**). Conversely, immune-related pathways, such as antigen processing and presentation, natural killer cell-mediated cytotoxicity, cytokine-receptor interactions, and the intestinal immune network for IgA production, were more active in the high-risk group.

### Construction of a nomogram and exploration of its clinical usefulness

**Figure 10:**
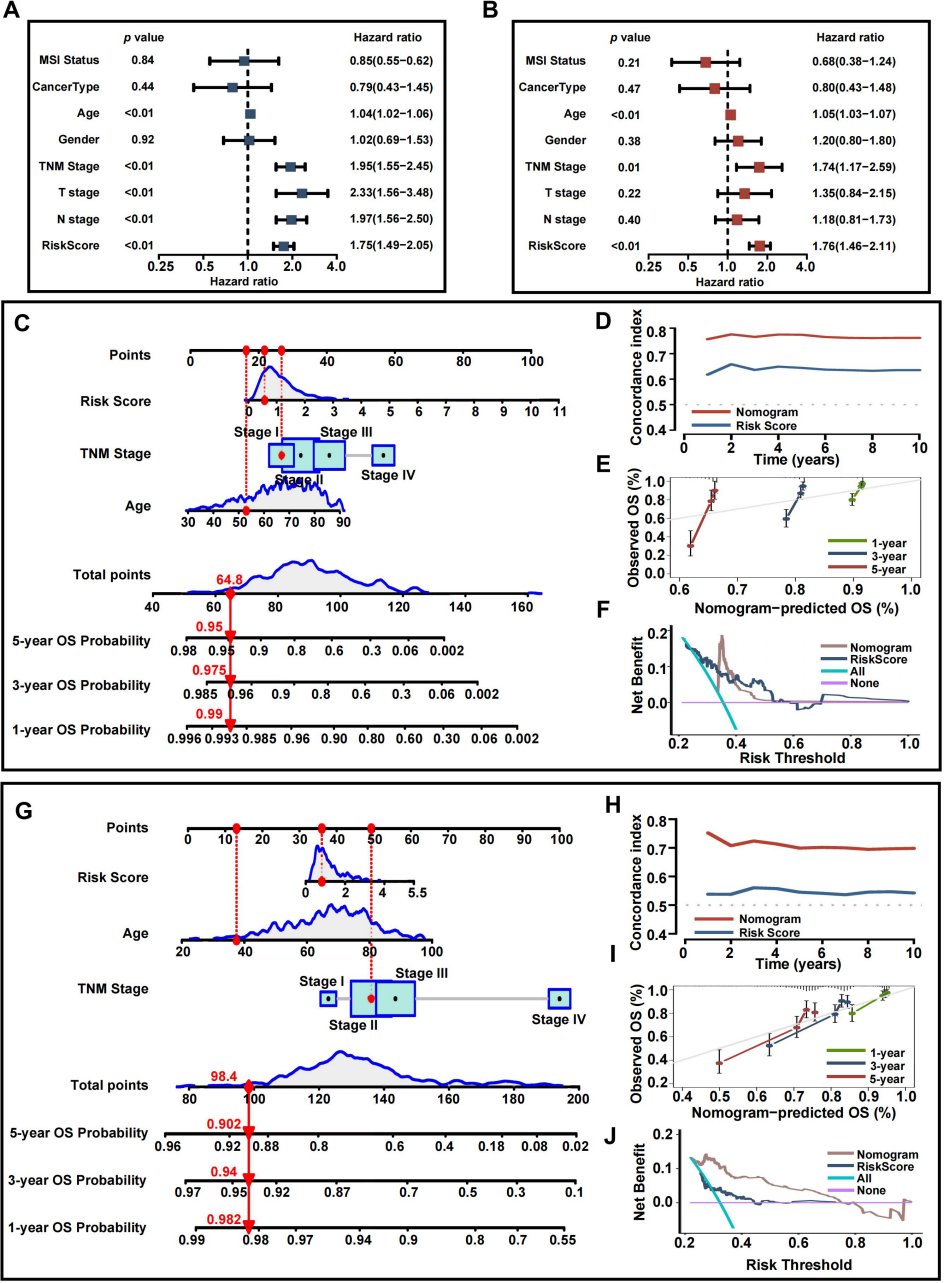
Development and assessment of the nomogram. **(A)** Univariate regression. **(B)** Multivariate regression of the clinicopathological indicators and gene signatures. **(C)** A comprehensive nomogram for predicting CRC patients’ survival probability in training set. **(D)** Time-dependant c-index plot for the nomogram and other clinical factors in training set. **(E)** Calibration curves of the nomogram at 1-, 3-, and 5-year intervals in training set. **(F)** DCA curves of the clinicopathological indicators and this nomogram in training set. **(G)** A comprehensive nomogram in validation set. **(H)** Time-dependant c-index plot in validation set. **(I)** Calibration curves in validation set. **(J)** DCA curves in validation set.

Given the strong correlation between a high risk score and increased mortality as well as poorer clinical outcomes, we explored whether the risk score could act as an independent prognostic factor for CRC patients. Both the CRRS and key clinicopathological indicators were assessed through univariate and multivariate Cox regression analyses (**Figures 10A, B**). After adjusting for potential confounders, multivariate analysis identified age (HR = 1.05, 95% CI: 1.03-1.07, P<0.01), TNM stage (HR = 1.74, 95% CI: 1.17-2.59, P=0.01), and risk score (HR = 1.76, 95% CI: 1.46-2.11, P<0.01) as significant factors. A total score was calculated for each patient by summing the points assigned to each prognostic variable (**Figure 10C**), with higher total scores associated with worse outcomes.

Time-dependent C-index curves, based on TCGA data, demonstrated that the nomogram outperformed the CRRS in terms of survival prediction accuracy (**Figure 10D**). Calibration plots for the TCGA cohort also indicated a strong agreement between predicted and actual overall survival (OS) (**Figure 10E**). To assess the clinical utility of the nomogram, a DCA was conducted, showing that the nomogram provided greater net benefit compared to the CRRS alone (**Figure 10F**). The nomogram’s predictive accuracy was further validated using the GEO cohort (**Figures 10G–J**).

### Relationships among coagulation-related signatures and TME revealed by single-cell analysis

**Figure 11:**
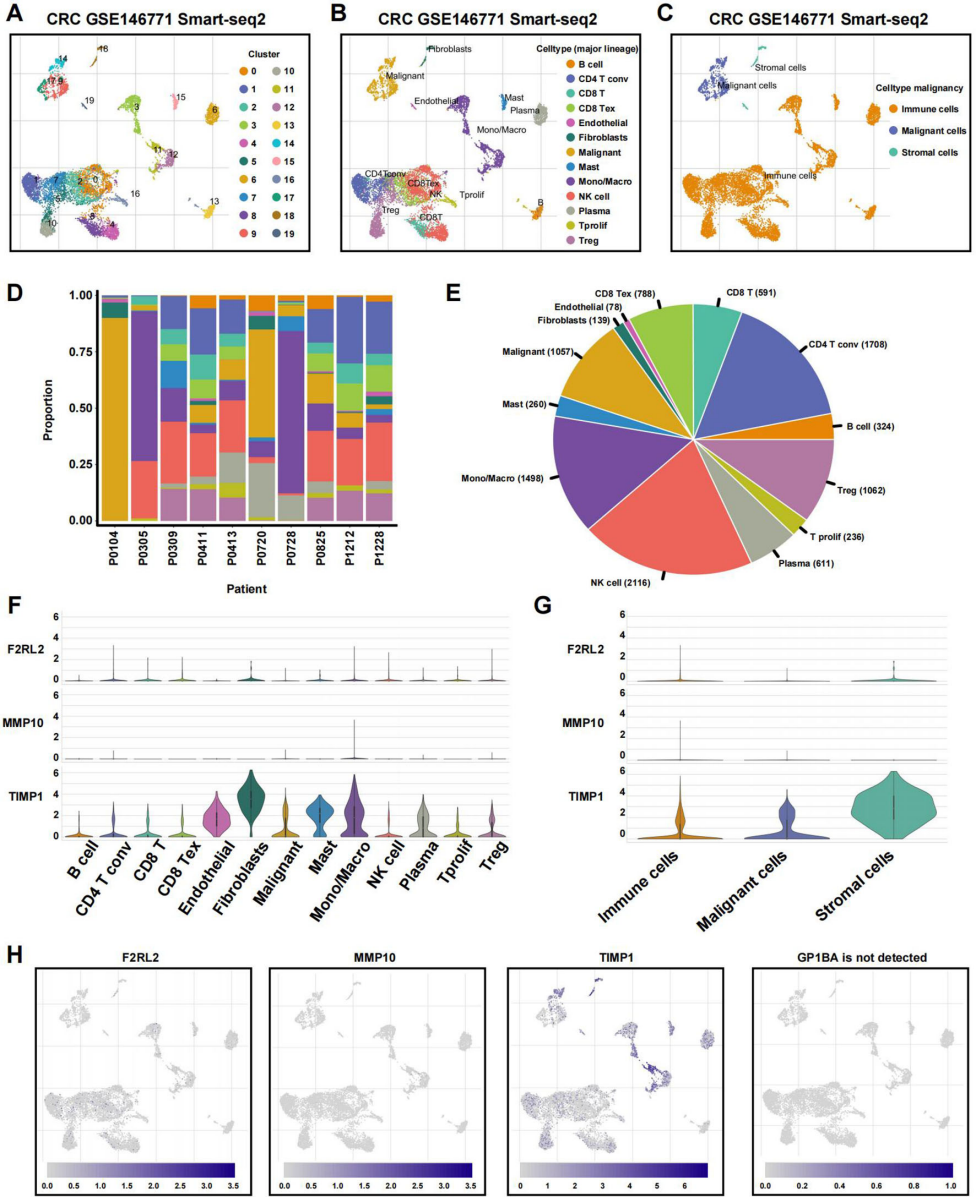
Single-cell profiles reveal CRGs expression patterns. **(A)** The identified cell clusters in colon cancer tissues based on the GSE146771 dataset. **(B)** The identified cell types in colon cancer tissues based on the GSE146771 dataset. **(C)** UMAP plot of immune, malignant and stromal cells from colon cancer scRNA-seq data. **(D, E)** Cell proportion in 10 colon cancer samples. **(F, G)** Violin plot for displaying the expression levels of CRGs in all cell types. **(H)** UMAP plots for visualizing the abundance distribution of CRGs.

To examine the associations between tumor microenvironment (TME) cell types and the expression of coagulation-related signatures, we analyzed GSE146771 CRC single-cell RNA sequencing (scRNA-seq) data. Using uniform manifold approximation and projection (UMAP)-based cell clustering following dimensionality reduction, we identified 19 distinct cell clusters (**Figure 11A**). These clusters were annotated based on lineage markers as B cells, conventional CD4+ T cells (CD4Tconv), CD8+ T cells, exhausted CD8+ T cells (CD8Tex), endothelial cells, fibroblasts, malignant cells, mast cells, monocytes or macrophages, NK cells, plasma cells, proliferating T cells (Tprolif), and regulatory T cells (Treg) (**Figure 11B**). The 19 clusters were then grouped into three main cell categories: immune cells, malignant cells, and stromal cells (**Figure 11C**).

**Figure 11D** displays the proportion of different cell types in each sample, and the percentage of absolute cell numbers across all CRC cases is visualized in **Figure 11E**. Our analysis revealed that TIMP1 was predominantly expressed across various cell types, particularly stromal cells, while MMP10 and F2RL2 exhibited low expression levels in both non-tumor and tumor cells (**Figures 11F–H**). Notably, GP1BA was undetectable at the single-cell level.

### TIMP1 facilitates the malignant proliferation of CRC cells

**Figure 12:**
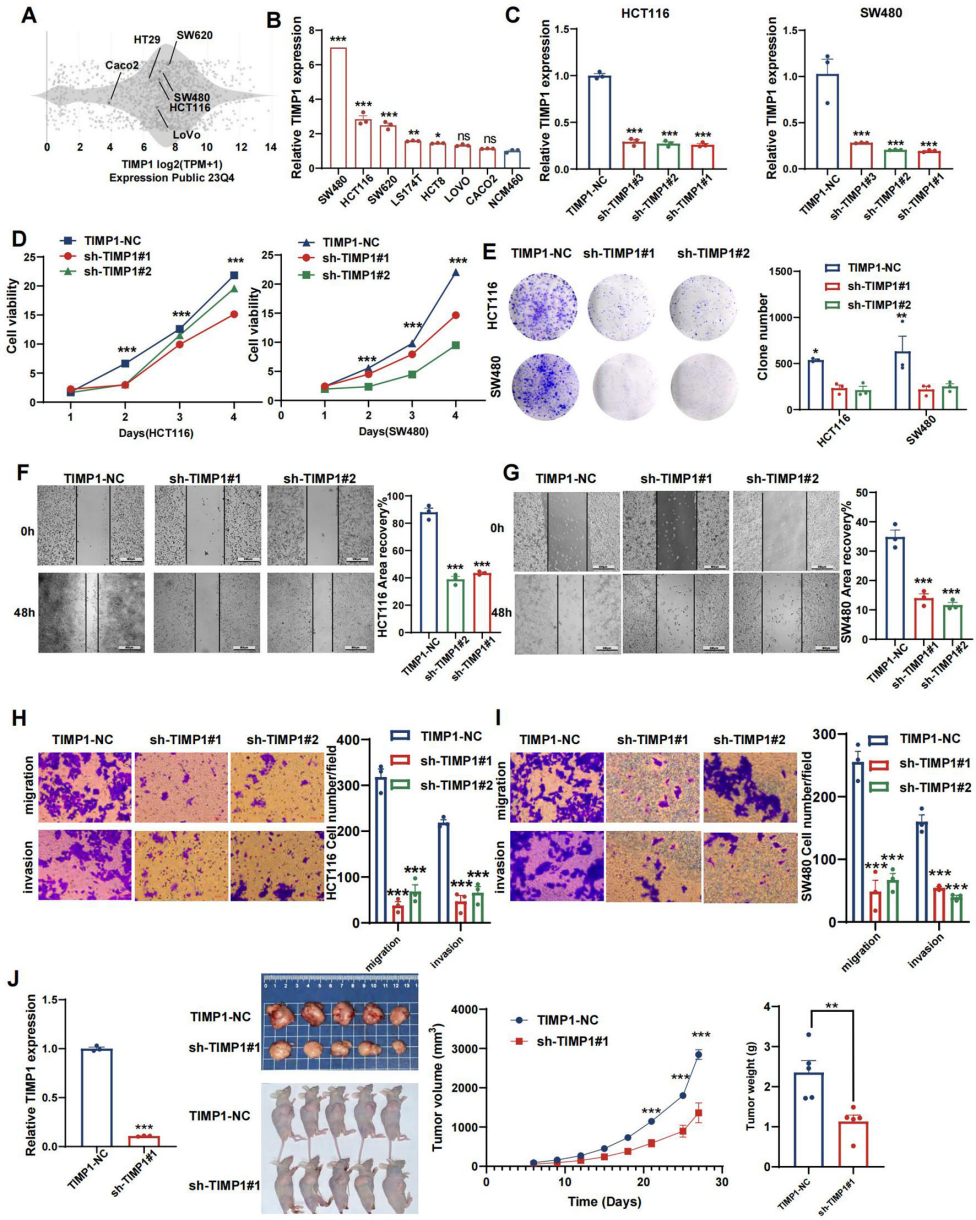
TIMP1 promotes malignant proliferation of CRC cells. **(A)** Distribution of mRNA expression across different cell lines obtained from the CCLE database. **(B)** qRT-PCR results of TIMP1 expression level in NCM460 and CRC cell lines. **(C)** Efficiency of TIMP1 knockdown in HCT116 and SW480 cells. **(D)** CCK-8 assay measuring cell viability in HCT116 and SW480 cells, respectively. **(E)** Colony formation assay assessing the colony-forming ability of HCT116 and SW480 cells. **(F, G)** Wound healing assay of migration in HCT116 **(F)** and SW480 **(G)** cells, respectively. **(H, I)** Transwell assay of migration and invasion in HCT116 **(H)** and SW480 **(I)** cells, respectively. **(J)** TIMP1 promotes CRC cell growth *in vivo*. (*p<0.05, **p<0.01, and ***p<0.001).

To experimentally validate our findings, we narrowed down the pool of coagulation- related signatures by constructing a protein-protein interaction (PPI) network. This involved querying the STRING database to identify hub genes among the four genes constituting the CRRS. As illustrated in **Supplementary Figure S9A**, our analysis revealed a complex interaction between TIMP1 and MMP10, suggesting their pivotal roles as central nodes in both coagulation and cancer pathways. The expression levels of TIMP1 and MMP10 at both the mRNA and protein levels, as well as their relationships with clinical outcomes, were explored in **Supplementary Figures S9B–Q**. According to previous studies, genes that are upregulated in tumors typically promote tumor growth and progression. Moreover, if these upregulated genes are inversely related to patients’ survival times, an increase in their expression levels will notably reduce patient survival rates (41). Therefore, we performed validation of TIMP1. A number of studies have explored the role of TIMP1 in CRC. Among these, one study found that overexpressed TIMP1 in right‐sided CRCs significantly correlates with a poor prognosis (42). However, the exact role of TIMP1 among all CRC patients remains unknown. At the RNA level, we utilized the CCLE dataset to acquire a gene expression matrix of CRC cell lines (**Figure 12A**). We verified the expression of TIMP1 in normal colon cells (NCM460) and CRC cells (SW480, HCT116, SW620, LS174T, HCT8, LoVo, and Caco2) by q-PCR assay (**Figure 12B**). TIMP1 was overexpressed in CRC cell lines. Next, we assessed SW480 and HCT116, which had the greatest levels of TIMP1 mRNA expression, as verified by RT-qPCR (**Figure 12C**). The CCK-8 assay was conducted to investigate the function of TIMP1 in CRC cell growth. Knockdown of TIMP1 inhibited the proliferation of SW480 and HCT116 cells (**Figure 12D**). Colony formation analysis demonstrated tumor cell growth to be weakened following TIMP1 knockdown (**Figure 12E**). In wound-healing and Transwell assays, migration, and invasion were reduced in shTIMP1 cell lines (**Figures 12F–I**).

### Reduced TIMP1 levels suppress tumor growth *in vivo*

We evaluated the impact of TIMP1 in a tumor xenograft model. Cells harboring either sh-control or sh-TIMP1 lentiviral vectors underwent subcutaneous transplantation into nude mice, with subsequent monitoring of tumor progression for 27 days. As depicted in **Figure 12J**, TIMP1 knockdown markedly attenuated tumor growth in the mice. Analysis of tumor volumes and weights revealed that tumors originating from shTIMP1-transduced cells were substantially smaller compared to those derived from sh-control transduced cells (P<0.001).

## Discussion

CRC represents a significant global health issue, ranking as the second leading cause of cancer-related mortality worldwide. The treatment options for CRC are varied, encompassing surgery, chemotherapy, radiation, targeted therapy, and immunotherapy, which can be applied either alone or in combination. Nevertheless, it is essential to acknowledge CRC’s inherent heterogeneity, evident not only in its anatomical locations but also in its clinical and pathologic characteristics, TME, and drug response profile. Heterogeneity presents considerable challenges in CRC management, emphasizing the necessity for personalized and targeted therapeutic strategies. Therefore, a stable classification system is essential for devising effective treatment plans and improving patient outcomes. Endothelial cells, platelets, and coagulation factors play a pivotal role in the coagulation process, which is intricately organized to maintain equilibrium between clot formation and bleeding (1). Over the past 10 years, a number of studies have supported the notion that coagulation was linked to infections, immune dysregulation, and malignancy (43, 44). According to theory, angiogenesis is a pivotal hallmark for cancer development, with a growing tumor requiring a significant blood supply (45). Cancer patients face a risk of developing coagulation abnormalities, especially cancer-associated thrombosis, with incidence much greater than that of the general population (46, 47). Additionally, it has been observed that cancer patients receiving chemotherapy or targeted therapy face an elevated risk of thrombotic events (46). Moreover, augmenting the immune response by employing immune checkpoint inhibitors can be associated with an increased mortality rate resulting from thrombotic complications (48, 49). Not only are tumor progression and prognosis associated with thrombotic problems, but such problems may also indicate an occult cancer (46). Some coagulation-related molecules are associated with tumor advancement as well as poorer prognosis (50). The close association of coagulation with tumorigenesis has prompted the functional evaluation of CRGs in TME, as a means by which to improve therapeutic strategies.

This study showed that CRSs and signatures effectively differentiated prognostic outcomes for CRC patients, despite significant tumor heterogeneity. The subtype classification was validated in two CRC cohorts and further extended to a pan-cancer analysis. The current clinical and pathologic staging system, which depended entirely on the anatomical extent of the tumor, did not adequately capture the biological variance observed in CRC patients. As a result, we developed a nomogram model incorporating age, TNM stage, and risk score to aid in assessing disease progression and providing personalized diagnosis. This nomogram has demonstrated excellent predictive performance. Furthermore, TIMP1 captured our focus due to its association with adverse CRC prognosis. Interfering TIMP1 expression in CRC cell lines HCT116 and SW480 produced a notable suppression of proliferation and migration by CRC cells.

While CMS represents a widely used gene expression-based system derived from 18 heterogeneous datasets, we explored whether our NMF-based classification using TCGA data could complement it (20). Our results showed that CRS subtype C1 was enriched in CMS2 tumors, whereas CRS-C3 largely overlapped with CMS4, which is known for stromal activation and poor prognosis. Notably, CRS-C3 also displayed strong immunosuppressive infiltration and worse survival outcomes. Beyond this alignment, the CRS system introduces additional stratification based on coagulation-related signatures, offering refined prognostic value and the potential to guide therapeutic decisions, particularly in predicting responses to chemotherapy and immunotherapy. Importantly, the CRS model demonstrated superior predictive performance (higher C-index) and clinical utility (higher net benefit in decision curve analysis) compared to CMS across both TCGA and GEO cohorts. These findings suggest that CRSs not only correlate with CMS but also provide complementary insights to better support personalized treatment strategies for CRC patients.

Factors related to the coagulation system within the TME contribute to conditions that favor tumor growth, metastasis, angiogenesis, and immune evasion (51–53). The interaction between the coagulation system and the immune system is critical in driving cancer progression (43). In our study, the C3 cluster and high-risk groups exhibited extensive immune cell infiltration, more non-tumor cell components, and reduced tumor purity, indicative of a more intense inflammatory and immune response. Furthermore, the high-risk group showed enrichment in immune activity-related signaling pathways, while cancer-related pathways were more prevalent in the low-risk group. Although tumor-infiltrating lymphocytes (TILs) are essential for improving survival outcomes, activated T-cells that fail to completely eradicate the tumor may become exhausted over time (54). This exhaustion, resulting from prolonged antigen exposure, leads to compromised cytotoxic T-cells, reducing their efficacy (55). Prior studies have shown that high levels of cytotoxic T-cells coupled with significant T-cell dysfunction can enhance tumor immune evasion, leading to severe dysfunction (33). Previous research has highlighted that high levels of cytotoxic T-cells, combined with significant T-cell dysfunction, can enhance tumor immune evasion, resulting in profound immune system dysfunction. Immune cell exhaustion, a phenomenon common in cancer and other diseases, drives tumor progression despite an active immune response (56). TIDE analysis, which evaluates immune dysfunction in tumors, revealed that cluster C3 and the high-risk group, both marked by elevated TIDE scores, are more likely to experience T-cell exhaustion (33). Our analysis of the TME revealed that immune infiltration was most extensive in the C3 and high-risk groups, including anti-tumor cells, Tregs, mast cells, and macrophages. Tregs within tumors suppress T-cell proliferation and activation, and together with tumor-associated macrophages, they form an immunosuppressive network that facilitates tumor immune evasion (57–59). Due to the potent immunosuppressive effects of Tregs and macrophages, the extensive immune infiltration observed in these groups does not guarantee effective tumor elimination. As a result, despite the increased presence of immune cells, immune escape may prevail because of functional impairment and the influence of suppressive cell populations, ultimately leading to a poor prognosis.

Our comprehensive analysis of differences in immunotherapy and chemotherapy patient responses across various molecular subtypes, along with our risk score model, has provided valuable insight into the coagulation system’s function in CRC and the therapeutic potential of coagulation-related models. TIDE and IPS scores predict the response rate of cancer patients to immunotherapy. A greater TIDE score relates to a higher risk of immune escape, and reduced immunotherapy benefit (33). IPS can be used to predict the response to immunotherapy agents PD1 and CTLA4 (60). The patients in the C1 cluster and low-risk group had lower TIDE scores and higher IPS scores than the patients in other groups, indicating that those patients may have an increased likelihood of a positive response to immunotherapy. The patients in C2 with the highest TIDE scores may show strong resistance to immunotherapy.

Additionally, we assessed the responses to various chemotherapeutic agents and targeted therapies across different risk score groups and molecular subtypes. Notably, despite showing increased resistance to immunotherapy, cluster C2 tumors exhibited heightened sensitivity to standard chemotherapies such as 5-FU, Oxaliplatin (FOLFOX), and Irinotecan. The analysis also identified potential new sensitive drugs for other subtypes. These results suggest that leveraging molecular subtypes and risk signatures could improve treatment outcomes. Therefore, a strategy that tailors the integration of immunotherapy, chemotherapy, and targeted therapy based on the innovative application of CRGs holds promise for personalized CRC treatment.

In addition, we also applied this molecular classification to other cancer types. We found that this classification enabled separation of most samples into three distinct clusters. In the majority of pan-cancer tissues, the immune microenvironment status of the molecular subtypes was similar to that of CRC. This was particularly true for hyper-coagulable cancers like bladder cancer and glioblastoma, with the classification showing excellent internal consistency and stability (61, 62). However, the pan-cancer analysis had certain limitations. First, the differences in the TME between subtypes within some cancers were not as pronounced as those observed in hypercoagulable cancers. Second, the survival outcomes and tailored treatment strategies across pan-cancers requires more in-depth investigation. While the coagulation-based classifier reproducibly stratifies multiple tumour entities, detailed survival outcomes and treatment implications across pan-cancer cohorts warrant dedicated future studies with harmonised clinical annotations.

The CRRS comprises four genes. Elevated expression of F2RL2 correlates with reduced risk and longer survival in colon adenocarcinoma (63). GP1BA-driven platelet–tumor interactions characterize the CRC macroenvironment and likely promote local immunosuppression and metastatic dissemination (64). Among these, MMP10 and TIMP1 form a functional interaction network. Subsequent in silico analyses revealed MMP10 overexpression in CRC, with higher protein levels correlating with improved patient survival. In this study, we found that TIMP1 was upregulated in CRC tissues, with higher expression levels correlating with a poor prognosis. As a tissue inhibitor of metalloproteinases, TIMP1 played a central role in our investigation. It serves as a key regulator of matrix metalloproteinases, controlling the turnover of the extracellular matrix (65, 66). Previous research has highlighted the importance of TIMP1 in the progression and metastasis of colon cancer, particularly through the FAK-PI3K/AKT and MAPK pathways (67). Importantly, TIMP1 knockdown has been shown to reduce migration, proliferation, and invasion in various tumor cell types (42, 65, 68). In this study, our *in vitro* functional assays further confirmed TIMP1’s role in promoting malignant cell proliferation, migration, and invasion (69–75). These results were confirmed *in vivo*, in which human CRC cells knocked-down for TIMP1 developed tumors at a slower pace than their wild-type controls. Targeting TIMP1 directly may be an approach for specific targeted treatment of CRC patients.

Our GO and KEGG enrichment analyses elucidated the underlying molecular pathways characteristic of each coagulation‐related subtype. Subtype C1 was predominantly associated with antigen processing and presentation via MHC class II, humoral immune responses, and complement–coagulation cascades, in keeping with its pronounced adaptive immune activity and favorable response to immunotherapy. Subtype C2 was distinguished by granulocyte and neutrophil chemotaxis, antimicrobial defense mechanisms, and matrix-degrading enzyme activity, indicative of an acute inflammatory microenvironment with active extracellular matrix remodeling that may promote tumor invasion. Subtype C3 exhibited marked enrichment for classical complement activation, platelet α-granule components, and ECM–receptor interactions, consistent with its pro-tumorigenic, immunosuppressive phenotype and poor clinical prognosis. These pathway‐level distinctions reinforce our findings on immune cell infiltration patterns, TMB/TIDE profiles, and patient outcomes, and they offer rationale for subtype‐specific therapeutic strategies—for example, modulation of MHC class II presentation in C1, protease inhibition in C2, and targeting of complement or platelet pathways in C3.

Our study faced certain constraints. First, the lack of transcriptomic data regarding immune checkpoint blockade therapy in CRC patients inhibited our ability to validate the discriminative efficacy of our predictive model (76). We intend to evaluate its accuracy as soon as ICB-related transcriptomic data for CRC patients become publicly available (77, 78). Second, the clinical data derived from public databases were incomplete, lacking essential clinical details, and were restricted to patients who have not received treatment. Consequently, the clinical utility of our risk model needs to be verified in a broader patient demographic. Third, functional validation was primarily conducted on TIMP1, although the risk signature consists of four genes. TIMP1 was prioritized due to its network centrality and clinical relevance; however, further studies are needed to elucidate the prognostic value of the full gene signature.

Overall, this novel subcategorization of CRC tumors and the related risk score and nomogram are of clinical interest for treatment of CRC and pan-cancer patients, with regard to both patient prognosis and therapeutic decision-making. The limitations of the model have been identified and further clinical investigations will be required to validate benefits of the subcategorization.

## Conclusions

In conclusion, our study delineates novel CRC subtypes based on CRGs, offering insights into prognosis and therapeutic strategies. The coagulation system’s influence on the TME and immune response underscores its significance in CRC progression and treatment. Our risk model and nomogram present promising tools for personalized treatment approaches. Furthermore, the applicability of this classification extends to other cancers, highlighting its potential for broad clinical impact. However, further validation and exploration are warranted to optimize its clinical utility and address existing limitations.

## Data Availability

The original contributions presented in the study are included in the article/**Supplementary Material**. Further inquiries can be directed to the corresponding author.

## Glossary

AUC: area under the ROC curve
CCK-8: cell counting kit-8
CD4Tconv: conventional CD4+ T cells
CD8Tex: exhausted CD8+ T cells
CGP: Cancer Genome Project
CMS: consensus molecular subtype
CAN: Copy number alteration
CRC: colorectal cancer
CRG: coagulation related genes
CRRS: coagulation-related risk score
CRSs: coagulation-related subtypes
CTLA-4: cytotoxic T-lymphocyte antigen 4
DCA: decision curve analysis
DEGs: differentially expressed genes
DFS: disease free survival
DSS: disease specific survival
FBS: fetal bovine serum
GEO: Gene Expression Omnibus
GSEA: gene set enrichment analysis
HLA: human leukocyte antigen
HPA: Human Protein Atlas
IHC: immunohistochemistry
IPS: immunophenoscore
LASSO: least absolute shrinkage and selection operator
MSI: microsatellite instability
NMF: nonnegative matrix factorization
OS: overall survival
PCA: Principal component analysis
GO: Gene Ontology
KEGG: Kyoto Encyclopedia of Genes and Genomes
PD-1: programmed cell death protein 1
PFS: progression free survival
PPI: protein-protein interaction
q-PCR: quantitative real-time PCR
ROC: receiver operating characteristic
scRNA-seq: single-cell RNA sequence
SNPs: Single nucleotide polymorphisms
SPF: specific pathogen-free
TCGA: The Cancer Genome Atlas
TCIA: the Cancer Immunome Atlas
TIDE: Tumor Immune Dysfunction and Exclusion
TILs: tumor infiltrating lymphocytes
TISCH: tumor immune single-cell hub
TME: tumor microenvironment
TMB: tumor mutation burden
TPM: transcripts per million
Tprolif: proliferating T cells
Treg: regulatory T cells
UMAP: uniform manifold approximation and projection
