# Supplementary material for: Identification of a coagulation-related classification and signature that predict disease heterogeneity for colorectal cancer and pan-cancer patients
Source: Front Immunol. 2025 Jul 24;16:1572701. doi: 10.3389/fimmu.2025.1572701 (PMC12328357; doi:10.3389/fimmu.2025.1572701)
Supplement: Supplementary file 1 [file DataSheet1.pdf]

Supplementary Materials

1.1 Supplementary Tables

Table S1. Catalog of coagulation-related genes.

Gene Name
A2M ACOX2 ADAM9 ANG ANXA1 APOA1 APOC1 APOC2 APOC3 ARF4 BMP1 C1QA C1R C1S C2 C3 C8A C8B C8G C9 CAPN2 CAPN5 CASP9 CD9 CFB CFD CFH CFI CLU COMP CPB2 CPN1 CPQ CRIP2 CSRP1 CTSB CTSE CTSH CTSK CTSO CTSV DCT DPP4 DUSP14 DUSP6 F10 F11 F12 F13B F2 F2RL2 F3 F8 F9 FBN1 FGA FGG FN1 FURIN FYN GDA GNB2 GNG12 GP1BA GP9 GSN HMGCS2 HNF4A HPN HRG HTRA1 ISCU ITGA2 ITGB3 ITIH1 KLF7 KLK8 KLKB1 LAMP2 LEFTY2 LGMN LRP1 LTA4H MAFF MASP2 MBL2 MEP1A MMP1 MMP10 MMP11 MMP14 MMP15 MMP2 MMP3 MMP7 MMP8 MMP9 MSRB2 MST1 OLR1 P2RY1 PDGFB PECAM1 PEF1 PF4 PLAT PLAU PLEK PLG PREP PROC PROS1 PROZ PRSS23 RABIF RAC1 RAPGEF3 RGN S100A1 S100A13 SERPINA1 SERPINB2 SERPINC1 SERPINE1 SERPING1 SH2B2 SIRT2 SPARC TF TFPI2 THBD THBS1 TIMP1 TIMP3 TMPRSS6 USP11 VWF WDR1 C5 PIK3R1 PLA2G4C C1QB PIK3R3 TBXA2R C4BPB PPP1CA SRC BDKRB1 CFHR1 MAPK1 ADCY4 ORAI1 PTGIR PRKACA MAPK12 MAPK14 AKT2 RAP1B PLAUR CFHR5 ARHGAP35 TLN2 F5 PIK3R5 AKT1 VASP F2RL3 PROCR ROCK2 VAMP8 ADCY2 GP6 MYL12A CFHR3 ADCY7 TBXAS1 PRKG1 C5AR1 ITPR2 SERPINF2 CD55 PLA2G4F LYN C3AR1 GNAS ACTB CR1L MYLK2 PPP1CB PIK3R6 JMJD7-PLA2G4B GNA13 ADCY9 PLCB4 ADCY3 C6 P2RX1 ITGB2 PRKACG PLCB1 ITPR1 PIK3CA FCGR2A GP5 C4B TLN1 VSIG4 NOS3 GNAI3 PRKCI PLA2G4A RASGRP1 RAP1A CFHR2 APBB1IP MYLK TFPI F7 RHOA FGB FERMT3 MYL12B PLCB3 MAPK3 ARHGEF12 ROCK1 C4BPA PLCG2 MAPK13 FCER1G COL1A1 ITGA2B GNAI2 SERPINA5 BTK PTGS1 C1QC MYLK3 C7 STIM1 SYK PPP1CC F13A1 ADCY8 CFHR4 PPP1R12A GNAQ VTN C4A KNG1 SERPIND1 PIK3R2 PIK3CB CR2 LCP2 RASGRP2 CR1 ITGB1 AKT3 ITPR3 MAPK11 ACTG1 ADCY5 MASP1 PIK3CG PRKCZ F2R PRKG2 PRKACB ADCY1 CD59 SNAP23 P2RY12 PLA2G4D COL1A2 ARHGEF1 BDKRB2 ITGAX COL3A1 PIK3CD PLCB2 GNAI1 MYLK4 ITGAM GUCY1A2

Table S2. Differentially expressed genes in coagulation-related subtypes.

Subtype	Differentially expressed genes
C1	SPHK1 SERPINE1 ITGA5 NNMT LGALS1 TYROBP CTSL CERCAM CAVIN1 LAPTM5 CD14 ANGPTL2 PLAU EHD2 ITGB2 TREM2 ADAMTS2 SPI1 TNC BGN FPR1 SOCS3 COL6A2 FCGR3A ALOX5AP TNFAIP6 FCER1G APOE VSIG4 OLFML2B CCL18 GPNMB COL1A1 SLC2A3 GAS1 COL6A1 CD163 NCF2 SPARC SRGN C1QC COL15A1 RAB31 AEBP1 CSF1R COL1A2 CD248 SERPING1 COL3A1 SPP1 BCL2A1 SFRP2 THBS2 C1QB FPR3 TYMP CD53 CCL2 OSM C1QA TAGLN COL5A2 POSTN CXCR4 COL6A3 EMILIN1 FSCN1 MMP1 FBLN2 MT2A TIMP2 MMP2 SULF1 CCN1 HLA-DPB1 PLEK CTHRC1 S100A8 PLCB4 CXCL8 FNDC1 IL1RN IGFBP5 DCN COL5A1 C1S COMP MMP9 HLA-DPA1 CHI3L1 HLA-DRA COL10A1 RAMP1 MARCO MMP3 GREM1 S100A9 HLA-DQA1 FN1 ANTXR1 MMP12 ISLR LUM HLA-DRB1 CNN1 CCL21 BST2 C3 TDGF1 HLA-DRB5 CXCL9 CXCL10 SFRP4 C10orf99 CDHR1 MEP1A HMGCS2 PLA2G2A LYZ FABP1 DES LEFTY1 REG1A
C2	MMP3 MMP10 MMP1 CXCL8 IL1B CXCL5 CXCL1 COMP MMP12 S100A9 REG1A REG1B LCN2 DMBT1 DES SPINK4 PLA2G2A REG3A PI3 REG4 TFF1 MUC2 JCHAIN PIGR
C3	COMP CILP PRELP TUBB6 SFRP2 AOC3 MFGE8 CAVIN1 TAGLN AEBP1 LMOD1 TNS1 HSPB7 PTGIS FLNA GAS1 TREM2 APOE OLFML2B FSTL3 SERPING1 MGP SSC5D ACTA2 TGFB3 EHD2 THBS4 CCDC80 FNDC1 MYL9 THBS2 BGN NOTCH3 CNN1 CERCAM MRC2 SFRP4 CLU COL6A2 C1R SPOCK1 HSPB8 CPXM2 MXRA8 EMILIN1 FBLN2 GPNMB ITGA11 HTRA3 CCN2 ITGB2 GPX3 MFAP5 NNMT COL1A1 ARL4C ISLR MYH11 LGALS1 DEPP1 PLN FBN1 COL1A2 PLIN4 DPYSL3 EFEMP1 COL8A1 MARCO COL10A1 COL6A1 TIMP2 VSIG4 ANGPTL2 FABP4 CHRDL1 TYROBP SULF1 FN1 CCN1 TPM2 SPARC COL11A1 LAPTM5 DES TIMP3 C1QC FLNC SPP1 HLA-DPB1 SPARCL1 ACKR1 COL3A1 COL5A1 COL6A3 FBLN1 APOC1 C3 SERPINE1 IGFBP5 CTHRC1 FCGR3A ACTG2 C1QA C1S COL5A2 ANTXR1 C1QB TNC ASPN MXRA5 CCL21 CHRDL2 GREM1 RAMP1 CCL19 POSTN MMP2 MMP9 MFAP4 MMP11 CCL18 APOD BST2 CCL20 MMP3 DUOX2 SPINK1 DUOXA2 LCN2 PHGR1 CEACAM7 C10orf99 OLFM4 PIGR CLCA1 SPINK4 TFF1 SLC26A3 MUC2 ITLN1

Table S3. Top 10 enriched GO terms per ontology for coagulation-related subtypes.

Subtype	Ontology	Description
C1	BP	extracellular matrix organization
		extracellular structure organization
		external encapsulating structure organization
		collagen fibril organization
		chemotaxis
		taxis
		myeloid leukocyte migration
		antigen processing and presentation of peptide antigen via MHC class II
		humoral immune response
		extracellular matrix organization
	CC	collagen-containing extracellular matrix
		collagen trimer
		endoplasmic reticulum lumen
		complex of collagen trimers
		fibrillar collagen trimer
		banded collagen fibril
		MHC class II protein complex
		endocytic vesicle
		MHC protein complex
		tertiary granule
	MF	extracellular matrix structural constituent
		extracellular matrix structural constituent conferring tensile strength
		collagen binding
		glycosaminoglycan binding
		extracellular matrix binding
		integrin binding
		proteoglycan binding
		platelet-derived growth factor binding
		MHC class II protein complex binding
		MHC protein complex binding
C2	BP	antimicrobial humoral response
		humoral immune response
		antimicrobial humoral immune response mediated by antimicrobial peptide
		neutrophil chemotaxis
		defense response to bacterium
		neutrophil migration

		collagen catabolic process
		granulocyte chemotaxis
		extracellular matrix disassembly
		granulocyte migration
	CC	IgA immunoglobulin complex
		immunoglobulin complex, circulating
		specific granule lumen
		secretory granule lumen
		cytoplasmic vesicle lumen
		vesicle lumen
		IgM immunoglobulin complex
		cell pole
		collagen-containing extracellular matrix
		specific granule
	MF	glycosaminoglycan binding
		peptidoglycan binding
		oligosaccharide binding
		CXCR chemokine receptor binding
		metalloendopeptidase activity
		chemokine activity
		serine-type endopeptidase activity
		metallopeptidase activity
		serine-type peptidase activity
		serine hydrolase activity
C3	BP	extracellular matrix organization
		extracellular structure organization
		external encapsulating structure organization
		collagen fibril organization
		cell-substrate adhesion
		ossification
		regulation of cell-substrate adhesion
		complement activation, classical pathway
		collagen metabolic process
		complement activation
	CC	collagen-containing extracellular matrix
		endoplasmic reticulum lumen
		collagen trimer
		basement membrane
		complex of collagen trimers
		fibrillar collagen trimer

		banded collagen fibril
		blood microparticle
		platelet alpha granule
		platelet alpha granule lumen
	MF	extracellular matrix structural constituent
		integrin binding
		extracellular matrix structural constituent conferring tensile strength
		glycosaminoglycan binding
		collagen binding
		heparin binding
		extracellular matrix binding
		sulfur compound binding
		fibronectin binding
		platelet-derived growth factor binding

BP, biological process; CC, cellular component; MF, molecular function.

Table S4. Top 30 enriched KEGG pathways for coagulation-related subtypes.

Subtype	Description
C1	Staphylococcus aureus infection
	Phagosome
	Rheumatoid arthritis
	Cytoskeleton in muscle cells
	ECM-receptor interaction
	Pertussis
	Leishmaniasis
	Protein digestion and absorption
	Complement and coagulation cascades
	Systemic lupus erythematosus
	Asthma
	Tuberculosis
	Intestinal immune network for IgA production
	Hematopoietic cell lineage
	AGE-RAGE signaling pathway in diabetic complications
	Herpes simplex virus 1 infection
	Allograft rejection
	IL-17 signaling pathway
	Focal adhesion
	Type I diabetes mellitus
	Graft-versus-host disease
	Viral myocarditis
	Viral protein interaction with cytokine and cytokine receptor
	Influenza A
	Chagas disease
	Amoebiasis
	Antigen processing and presentation
	Autoimmune thyroid disease
	Proteoglycans in cancer
	PI3K-Akt signaling pathway
C2	IL-17 signaling pathway
	Rheumatoid arthritis
	Lipid and atherosclerosis
	Amoebiasis
	TNF signaling pathway
	Malaria
	Legionellosis
	Pertussis
	Coronavirus disease - COVID-19
	Viral protein interaction with cytokine and cytokine receptor
	NF-kappa B signaling pathway

	Cytokine-cytokine receptor interaction
	Alcoholic liver disease
	Bladder cancer
	NOD-like receptor signaling pathway
	Chemokine signaling pathway
	Epithelial cell signaling in Helicobacter pylori infection
	AGE-RAGE signaling pathway in diabetic complications
	Chagas disease
	Toll-like receptor signaling pathway
	Yersinia infection
	Gastric cancer
	Non-alcoholic fatty liver disease
	Influenza A
	Kaposi sarcoma-associated herpesvirus infection
	alpha-Linolenic acid metabolism
	Transcriptional misregulation in cancer
	Pathogenic Escherichia coli infection
	Linoleic acid metabolism
	Antifolate resistance
C3	Cytoskeleton in muscle cells
	ECM-receptor interaction
	Complement and coagulation cascades
	Protein digestion and absorption
	Focal adhesion
	Phagosome
	Staphylococcus aureus infection
	Pertussis
	Malaria
	Human papillomavirus infection
	AGE-RAGE signaling pathway in diabetic complications
	Amoebiasis
	Systemic lupus erythematosus
	PI3K-Akt signaling pathway
	Chagas disease
	Leishmaniasis
	Relaxin signaling pathway
	Rheumatoid arthritis
	Dilated cardiomyopathy
	Proteoglycans in cancer
	Diabetic cardiomyopathy
	Coronavirus disease - COVID-19
	Tuberculosis
	Vascular smooth muscle contraction

	Apelin signaling pathway
	Motor proteins
	IL-17 signaling pathway
	Hypertrophic cardiomyopathy
	Viral protein interaction with cytokine and cytokine receptor
	Leukocyte transendothelial migration

Table S5. Primers sequences used in qRT-PCR.

Gene	Forward primer (5’ → 3’)	Reverse primer (5’ → 3’)
TIMP1	CTGTTGTTGCTGTGGCTGATAG	ATAACGCTGGTATAAGGTGGTCTG

Table S6. Sequences of shRNAs for TIMP1.

shRNA	Sequence
TIMP1 shRNA1	5'-GAAGTCAACCAGACCACCTTA-3'
TIMP1 shRNA2	5'-ACAGACGGCCTTCTGCAATTC-3'
TIMP1 shRNA3	5'-GCACAGTGTTTCCCTGTTTAT-3'
Negative control sense	5'-TTCTCCGAACGTGTCACGT-3'

Supplementary Figures

1.1 Supplementary Figures

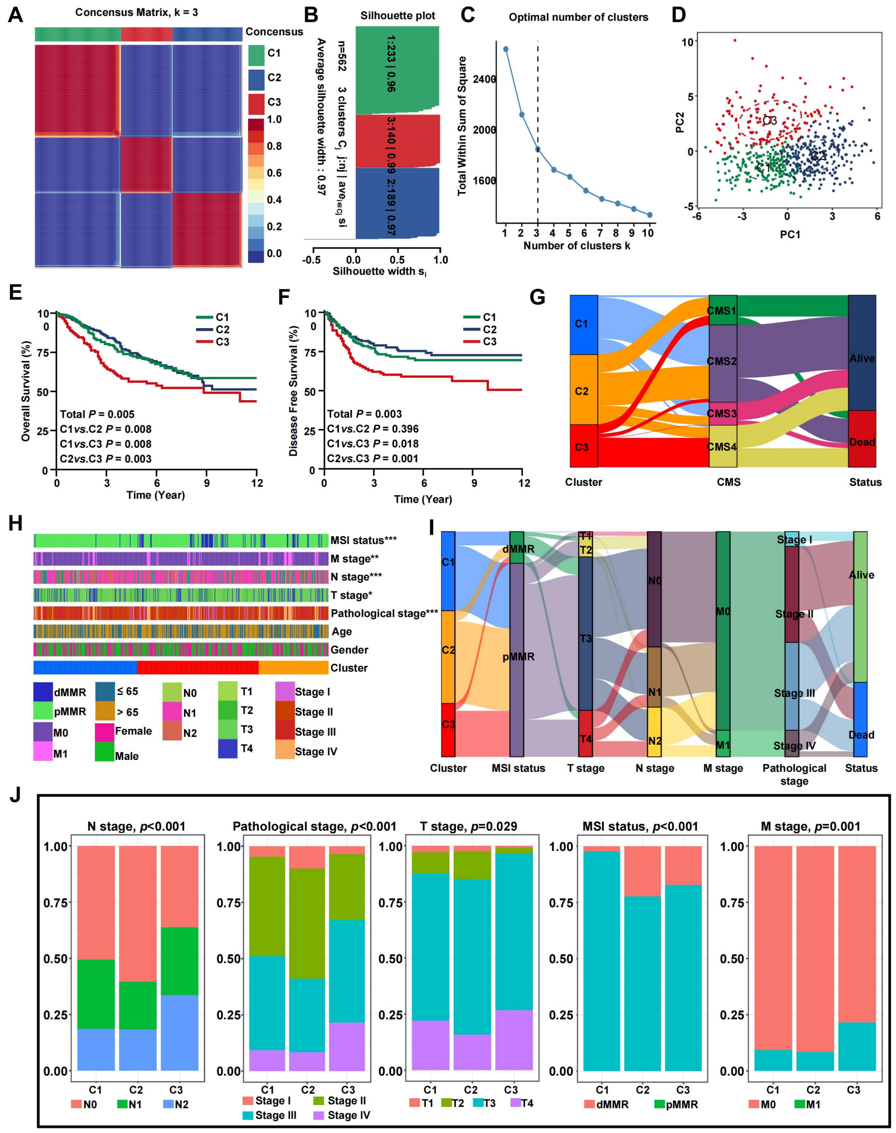

Figure S1. Consensus clustering of coagulation-related genes in validation cohort. (A) The consensus clustering heat map visualizes the degree of segmentation. (B) The average silhouette width represents the coherence of clusters. (C) The optimal number of clusters. (D) Principal component analysis plots. (E-F) Kaplan-Meier overall survival, and disease-specific survival. (G) The correspondence between CRS, CMS and survival status. (H) Heatmap presenting the clinicopathologic features of these subtypes. (I) Sankey diagram showing the relationship between CRS, MSI status, T stage, N stage, TNM stage and status. (J) The distribution characteristics of different clinicopathological factors in three subtypes. (*p<0.05, **p<0.01, and ***p<0.001).

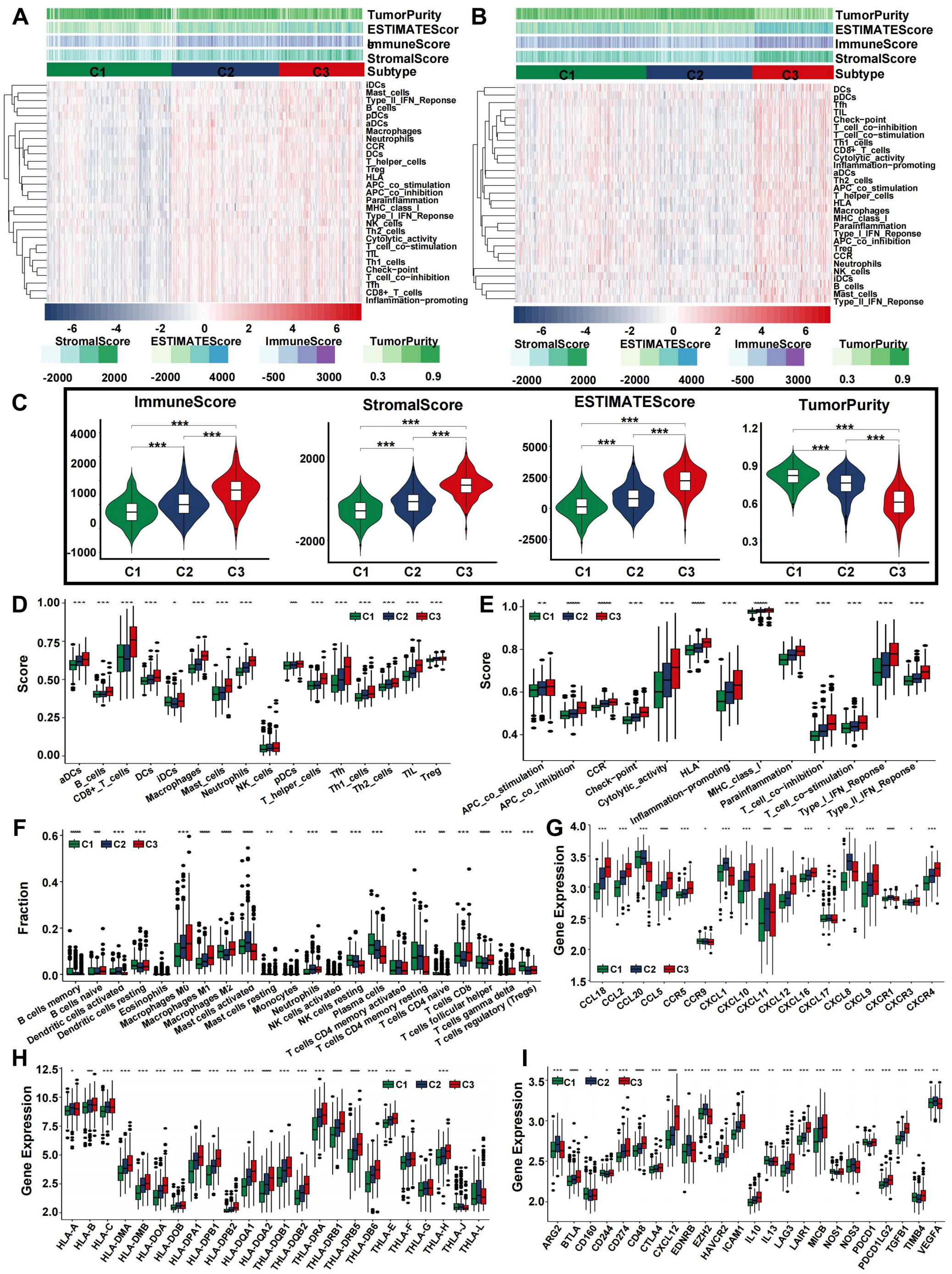

Figure S2. Immune landscape of CRS in the validation cohort.(A-B) The heatmaps show immune score, stromal score, ESTIMATE score, and tumor purity in the training cohort and validation cohort. (C) The violin plots display the immune score, stromal score, estimate score, and tumor purity score in the validation cohort.(D-E) Immune cell infiltration (D) or functions (E) in the C1, C2 and C3 groups in the validation cohort. (F) Comparison of immune cell infiltration proportions in C1, C2, and C3 using the CIBERSORT method in validation cohort. (G-H) Boxplots representing the differential expression of chemokines (G) , HLA gene sets (H), and immune checkpoints (I). (*p<0.05, **p<0.01, and ***p<0.001).

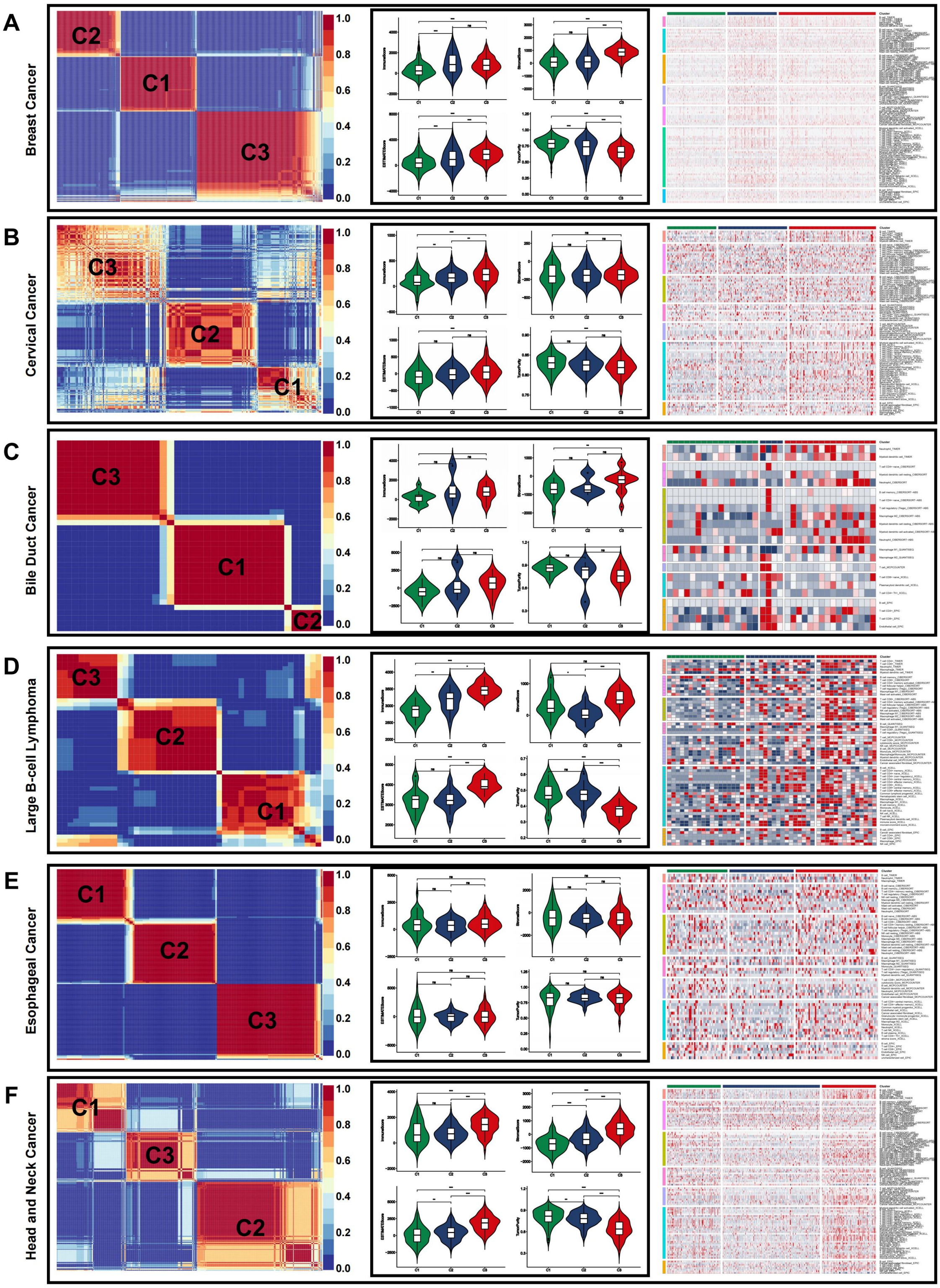

Figure S3. Immune microenvironment of CRSs in pan-cancer. (A-F) The immune microenvironment in representative cancer: breast cancer, cervical cancer, bile duct cancer, large B-cell lymphoma, esophageal cancer, and head and neck cancer.

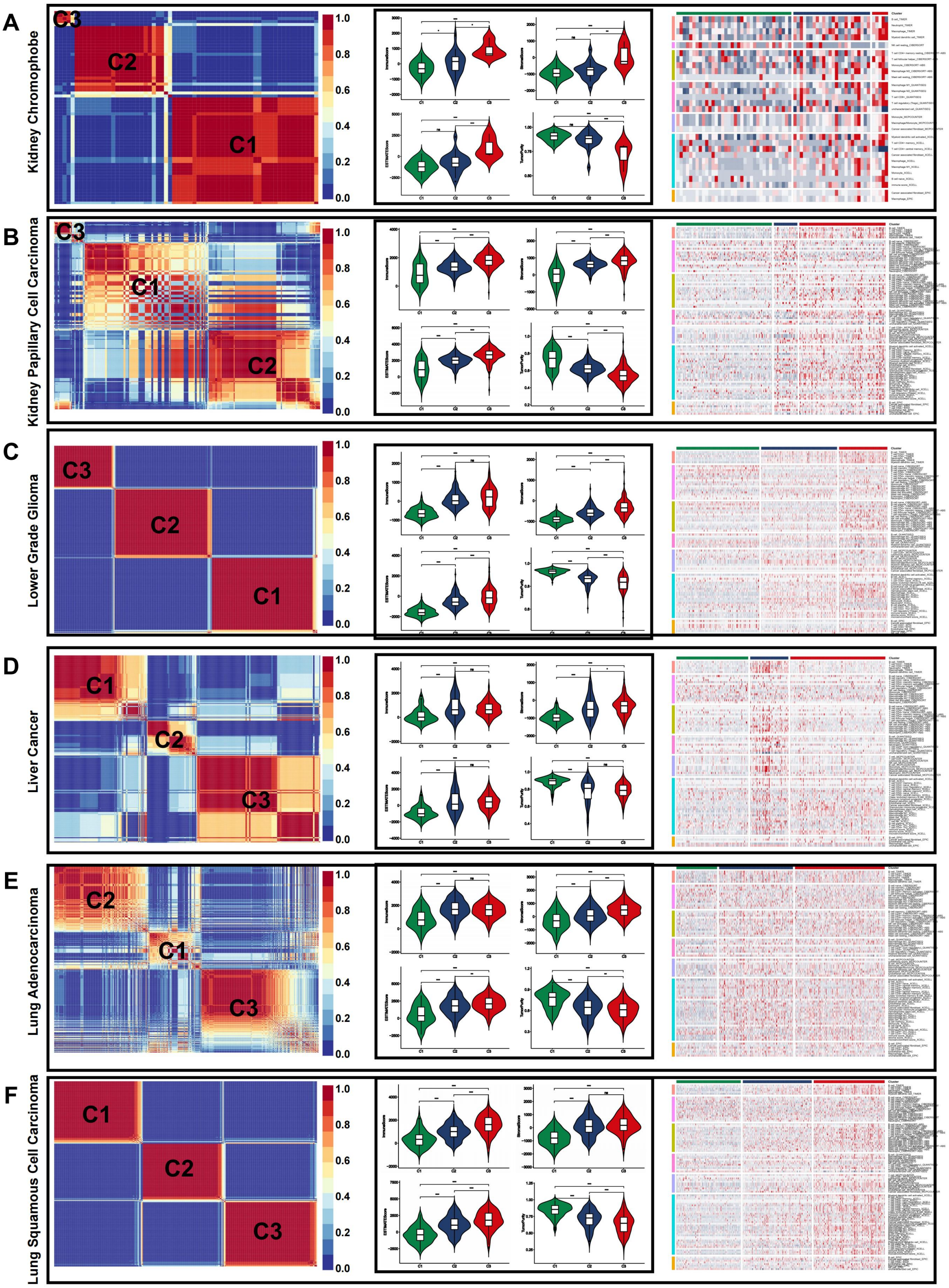

Figure S4. Immune microenvironment of CRSs in pan-cancer. (A-F) The immune microenvironment in representative cancer: kidney chromophobe, kidney papillary cell carcinoma, lower grade glioma, liver cancer, lung adenocarcinoma, and lung squamous cell carcinoma.

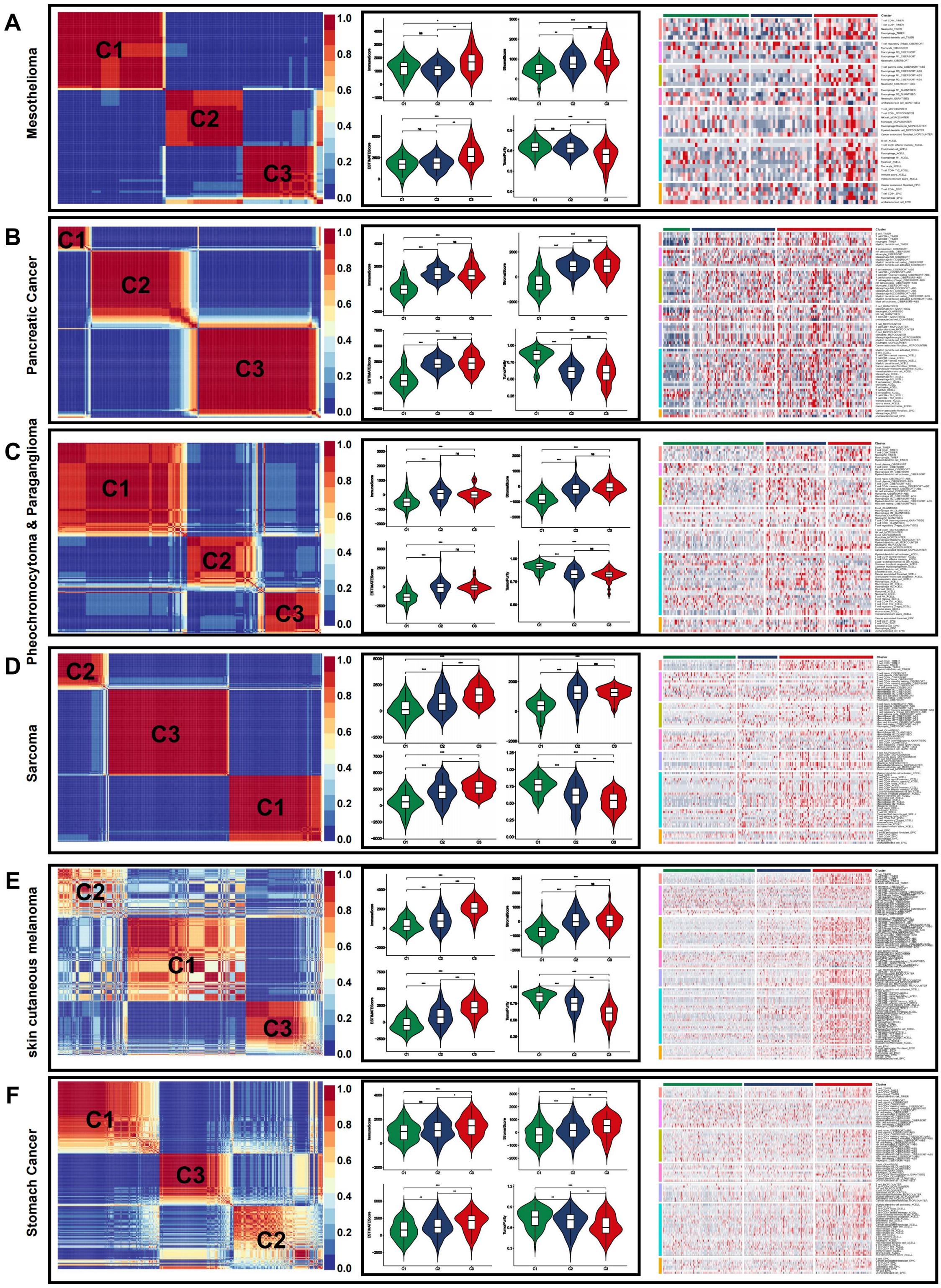

Figure S5. Immune microenvironment of CRSs in pan-cancer. (A-F) The immune microenvironment in representative cancer: mesothelioma, pancreatic cancer, pheochromocytoma & paraganglioma, sarcoma, skin cutaneous melanoma, and stomach cancer.

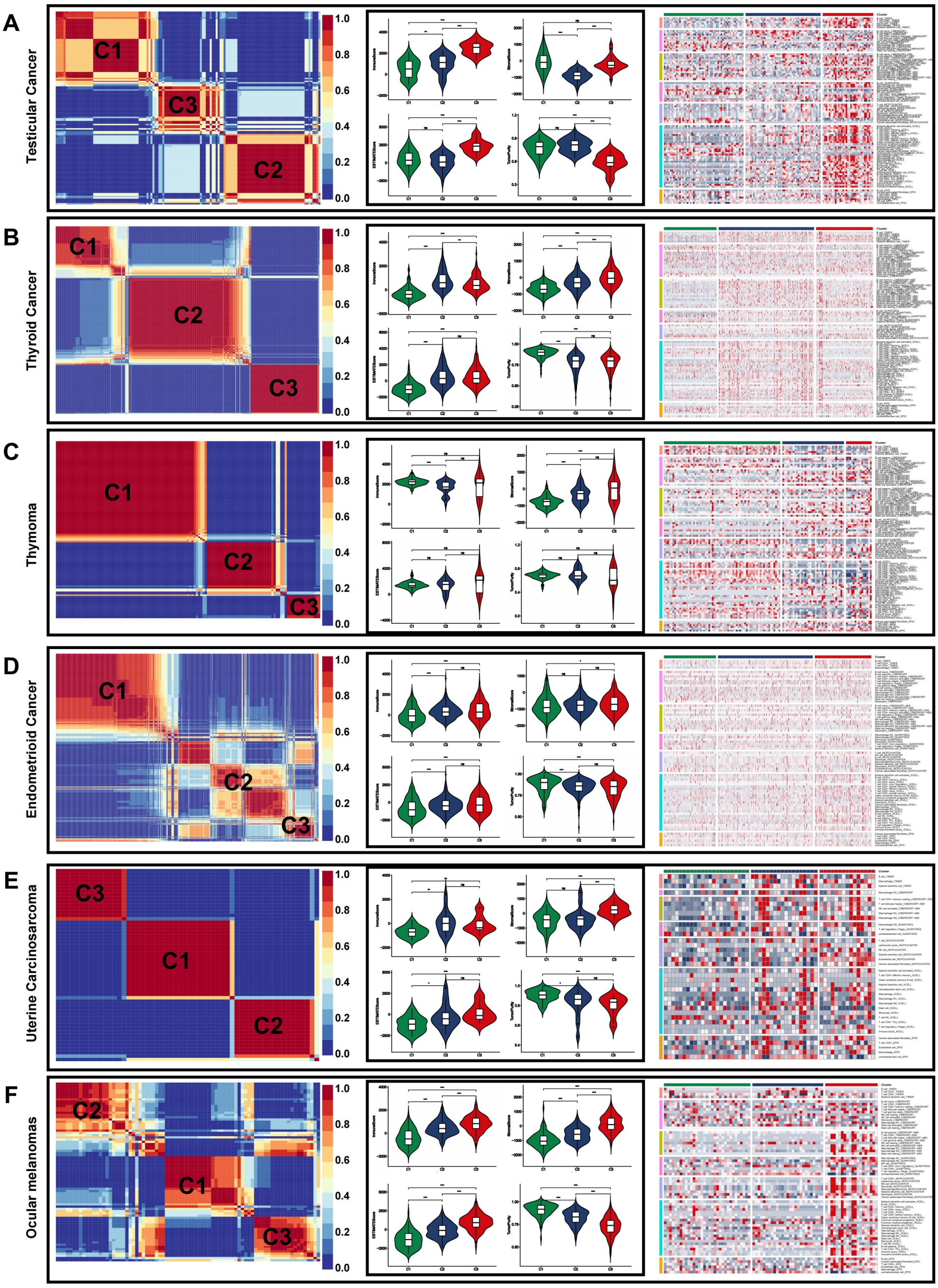

Figure S6. Immune microenvironment of CRSs in pan-cancer. (A-F) The immune microenvironment in representative cancer: testicular cancer, thyroid cancer, thymoma, endometrioid cancer, uterine carcinosarcoma, and ocular melanomas.

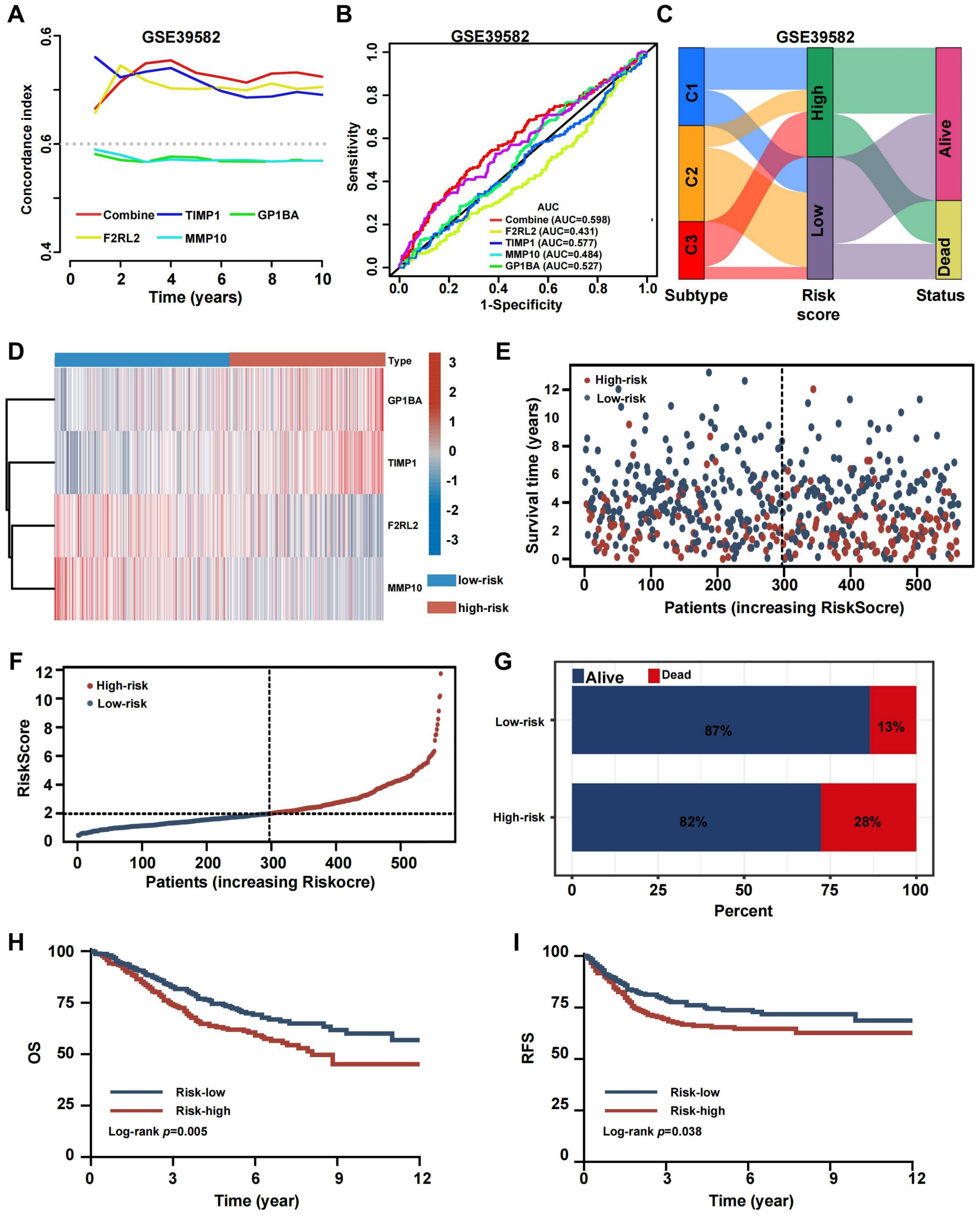

Figure S7. Construction and validation of the coagulation-related prognostic signature in validation cohort. (A) Time-dependent C-index plot for the risk score and individual genes. (B) The AUC assess the accuracy of the risk score. (C) Sankey plot summarized the relationships among the clusters, risk score and survival status. (D) Heatmaps of the prognostic signature in the validation cohort. (E-G) Survival status and risk score of the two risk groups. (H-I) Kaplan-Meier OS (H), and RFS (I) curves for patients with high- or low-risk scores in validation cohort.

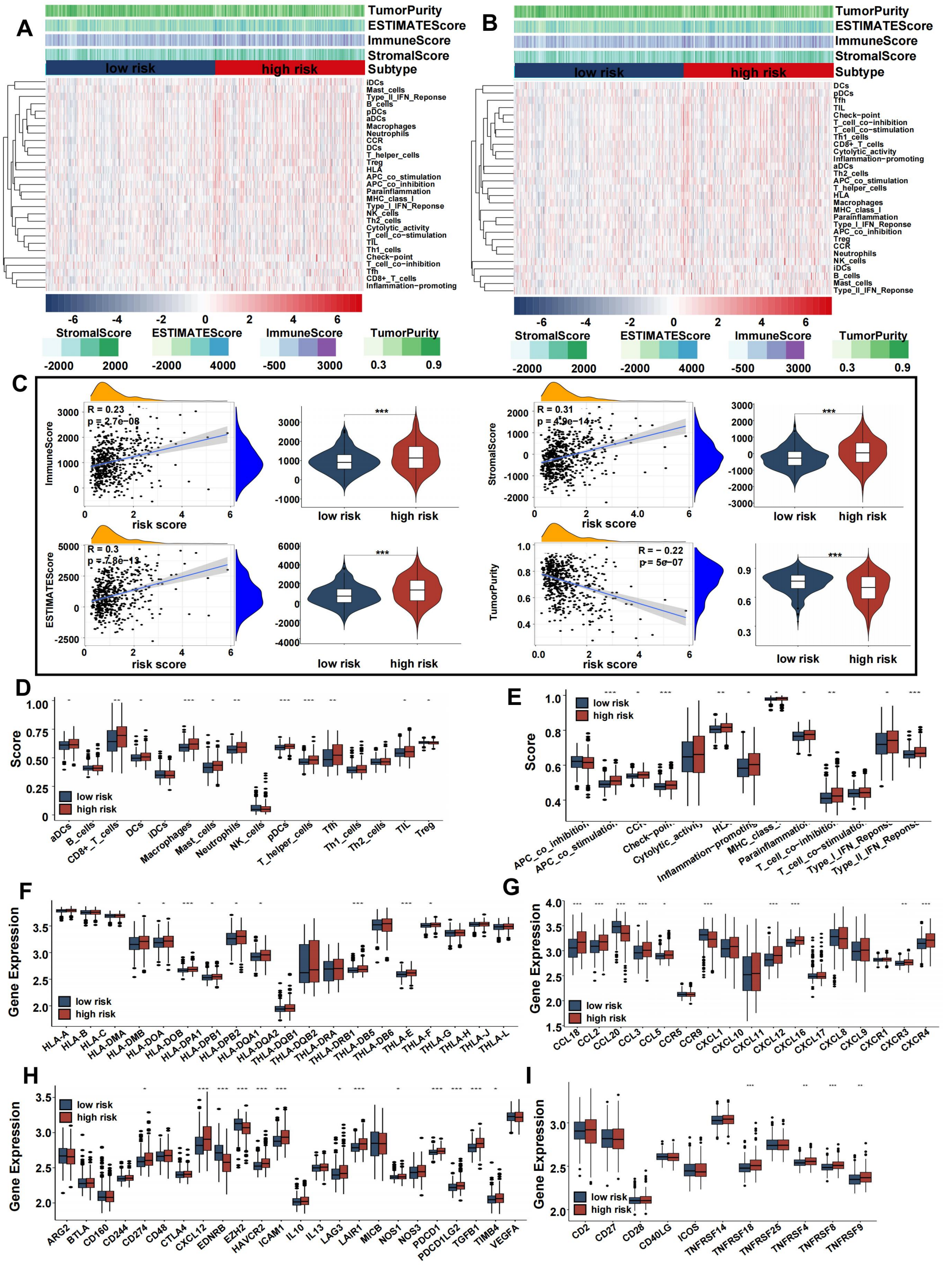

Figure S8. Immune association of coagulation risk score. (A-B) The heatmaps show immune score, stromal score, ESTIMATE score, and tumor purity in the training cohort and validation cohort. (C) Violin plots comparing the immune score, stromal score, ESTIMATE score, and tumor purity between high- and low-risk groups.(D-E) Boxplots comparing scores for immune cell types and immune-related functions between high- and low-risk groups. (F-I) Boxplots representing the differential expression of HLA gene sets (F), chemokines (G), immune checkpoints (H), and tumor necrosis factor superfamily between high- and low-risk groups (I). (*p<0.05, **p<0.01, and ***p<0.001).

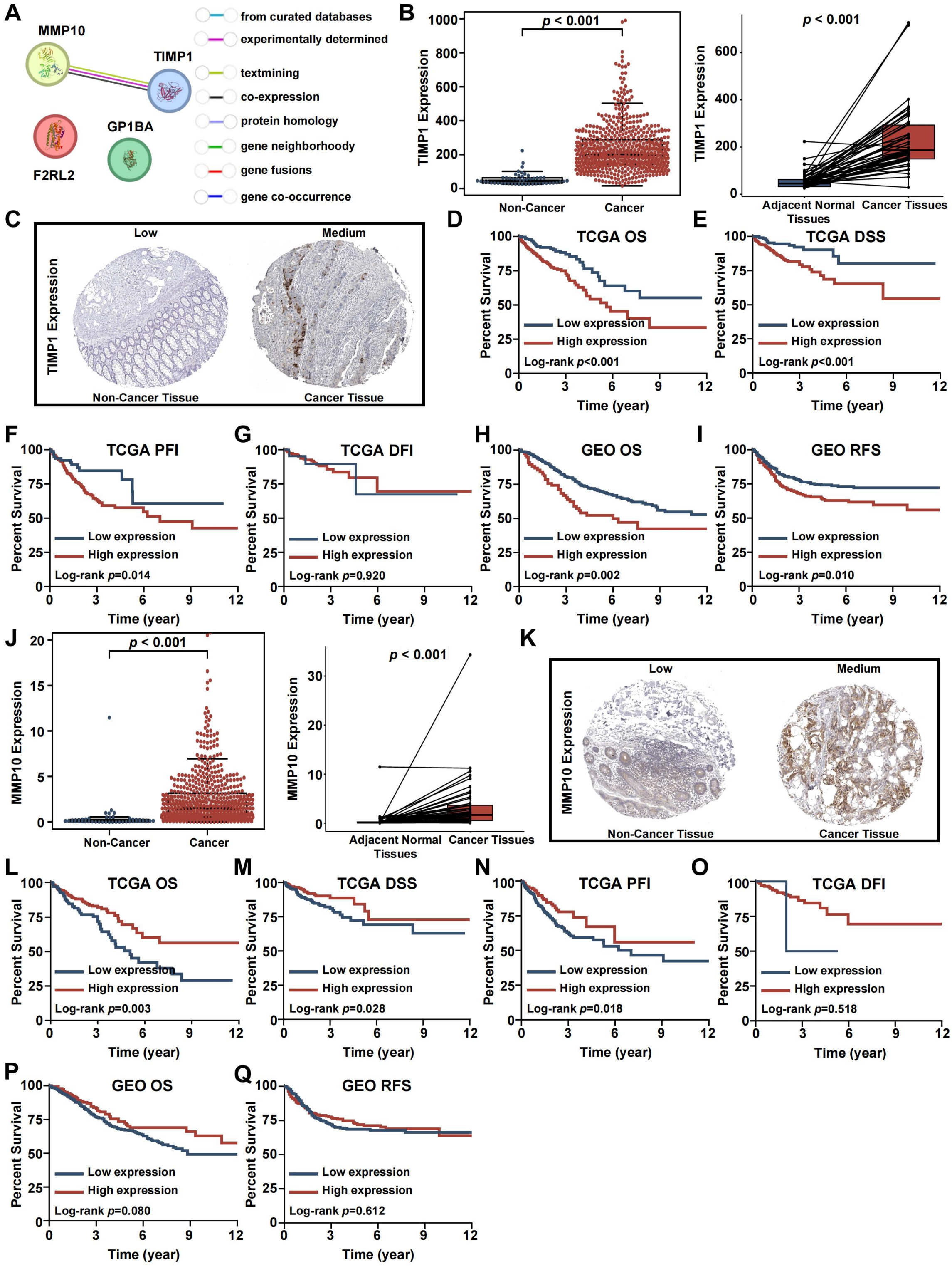

Figure S9. Identification of hub coagulation-related prognostic signature. (A) Protein–protein interactions among 4 coagulation-related genes. (B) Differential analysis for TIMP1. (C) The expression of TIMP1 protein in colorectal cancer tissues. (D-G) Kaplan-Meier analysis of the association between the expression levels of TIMP1 and prognosis, including OS (D), DSS (E), PFI (F), and DFI (G) in training cohort. (H-I) Kaplan-Meier analysis of the association between the expression levels of TIMP1 and prognosis, including OS (H), and RFS (I) in validation cohort. (J)

Differential analysis for MMP10. (K) Theexpression of TMMP10 protein in colorectal cancer tissues. (L-O) Kaplan-Meier analysis of the association between the expression levels of MMP10 and prognosis, including OS (L), DSS (M), PFI (N), and DFI (O) in training cohort. (P-Q) Kaplan-Meier analysis of the association between the expression levels of TIMP1 and prognosis, including OS (P), and RFS (Q) in validation cohort.
